# Anionic azo dyes removal from water using amine-functionalized cobalt–iron oxide nanoparticles: a comparative time-dependent study and structural optimization towards the removal mechanism†

**DOI:** 10.1039/c9ra07686g

**Published:** 2020-01-03

**Authors:** Qurrat-ul-Ain, Sumaira Khurshid, Zarnab Gul, Jaweria Khatoon, Muhammad Raza Shah, Irum Hamid, Iffat Abdul Tawab Khan, Fariha Aslam

**Affiliations:** Department of Chemistry, University of Karachi Karachi-75270 Pakistan qurrat_chem@uok.edu.pk +92 21 99261330 +92 21 99261300; Department of Chemistry, Federal Urdu University of Arts, Science and Technology, Gulshan-e-Iqbal Campus Karachi-75300 Pakistan; H. E. J. Research Institute of Chemistry, ICCBS, University of Karachi Karachi 75270 Pakistan raza.shah@iccs.edu

## Abstract

The current study is aimed at synthesizing and characterizing magnetic cobalt–iron oxide nanoparticles (CoFeNPs) functionalized with two different amino reagents, hydrazine and dodecylamine, resulting in CoFeNPs1 and CoFeNPs2, respectively. Both types of cobalt–ferrite nanoparticles were investigated for the removal of six different negatively charged azoic dyes (Amaranth, Acid Orange 7, Naphthol Blue Black, Reactive Orange 16, Acid Orange 52 and Reactive Red-P2B) from water, and their removal efficiency was compared as a function of different factors such as time, type of anchored amine, size of CoFeNPs and structure of the dye. CoFeNPs were successfully characterized by FT-IR spectra, AFM, SEM-EDS, surface charge (ζ-potential) and thermal analysis. CoFeNPs1 revealed 44.5–82.1% dye removal at equilibrium (attained within 28–115 min) with an adsorptive capacity (*q*_e_) of 5.4–13.5 mg g^−1^ observed under unoptimized conditions (temp. 30 °C, adsorbent dose 0.67 g L^−1^, pH 6, dye concentration 20 μmol L^−1^). Use of CoFeNPs2 significantly enhanced the removal of each dye (percent dye removal 68.0–98.9%, *q*_e_ 6.6–23.5 mg g^−1^) compared to CoFeNPs1 under similar conditions. From a comparative structural study, a larger size, more complex structure, hydrophobic character and greater number of phenyl SO_3_^−^ groups among the tested dyes facilitated their removal by CoFeNPs2, while all of these structural factors were negatively related to dye removal by CoFeNPs1. CoFeNPs2 showed some dye aggregation along with adsorption, while in the case of CoFeNPs1, only adsorption was observed as confirmed by FT-IR and UV-visible spectral studies. Dye removal data in all cases was in best compliance with pseudo-second order kinetics in comparison to pseudo-first order or the Elovich model, where film diffusion was a dominant phenomenon compared to intra-particle diffusion. Adsorption isotherms, thermodynamics and reusability of the CoFeNPs were studied selecting Reactive Orange 16. Adsorption equilibrium was best fitted to the Langmuir isotherm. Δ*G*° and Δ*H*° indicated spontaneous and exothermic adsorption. Amine-functionalized CoFeNPs are recommended as potential cost-effective adsorbents with excellent reusability that could be applied efficiently for rapid and selective dye removal from textile effluents considering the size, structure, charge and number of S atoms in the target azo dyes.

## Abstract 2

Efficient and selective removal of azo dyes from water by amine-functionalized-CoFe_2_O_4_ nanoparticles reliant on structural features such as size, charge, hydrophobicity/hydrophilicity, and S/C atoms.

## Graphical abstract

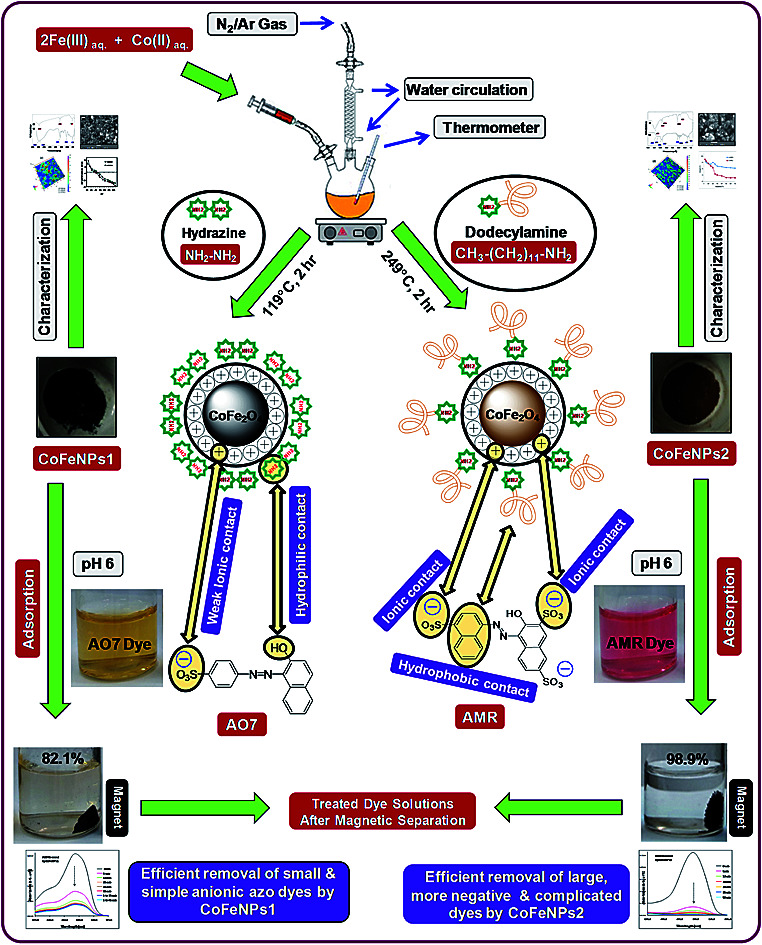

## Introduction

A wide range of robust applications of nanotechnological materials have attracted researchers towards this field.^1^ Properties and applications of nanomaterials are usually governed by shape, size, chemical composition and overall molecular structure. Magnetic nanoparticles (NPs) are one of the most fascinating nanomaterials with versatile applications, particularly in magnetic data storage, magnetic resonance imaging, magnetic fluids, biotechnology/biomedicine, high performance inductors, catalysis and environmental remediation.^2–4^

The environmental water pollution caused by inorganics (metal ions), and synthetic organic compounds or their degradation products, such as phenols, organochlorines, polycyclic aromatic hydrocarbon, pesticides, polychlorinated biphenyl, polymers and synthetic dyes, is a great challenge of the modern world due to their persistent nature and ultimate detrimental effects on humans and other living organisms.^5^ Synthetic dyes are frequently used (0.7 million tons of about 100 000 different kinds per year) in different industries, including the food, cosmetics, leather, pharmaceutical, paper, plastic and textile industries.^6–8^ Azo dyes, usually anionic, are amongst the most detrimental types of dyes because of their high thermal, optical and physico-chemical stability, attributed to their stable chemical composition involving aromatic rings and azoic linkages. The unrestrained discharge of these dyes into water reservoirs leads to serious environmental problems, mainly intrinsic toxicity, carcinogenic effects, skin sensitization, mutagenic effects and reduction of sunlight in aquatic environments, posing a serious threat to aquatic organisms and humans.^6,9–11^ Therefore, elimination of these dyes is necessary before the discharge of industrial effluents into natural streams, and so it has become a hot topic in current material and environmental research.

Dye removal from effluents is usually executed through chemical (Fenton's reagents, photochemical methods, the sodium hypochlorite method, electrochemical destruction, *etc.*), biological (decolorization by living/dead microbial biomass, white-rot fungi, anaerobic bioremediation, *etc.*) and physical (ion exchange, electro-kinetic coagulation, nanofiltration/membrane filtration, irradiation, adsorption, *etc.*) means.^12^ Adsorption is deemed as one of the most efficient, inexpensive and simple techniques for water purification.^8^ Many kinds of absorbents are commercially available for specific pollutant removal applications.^5,13,14^ For example, activated carbon (AC) is frequently applied to remove heavy metals and other pollutants owing to its high surface area and meso- and micro-porosity. Nevertheless, AC is conventionally prepared from non-renewable coal, which raises the cost and results in difficulty during disposal and regeneration. The adsorbents that are commercially available may also suffer from the problems of low adsorption capacity or long equilibrium time.^5^ Therefore, other more eco-friendly and cost-effective materials are demanded with efficient adsorption properties. Magnetic nanoparticles, particularly magnetic iron oxides, have also shown their valuable role as adsorbents in environmental remediation, particularly against heavy metal ions, dyes and other inorganic and organic compounds. This application of magnetic NPs is associated with their dominant features of reduced size (high surface area), low cost, easy and quick magnetic separation, fast reactivity, high environmental stability and adsorption capacity, easy surface functionalization and low biotoxicity.^2,15^

Magnetic nanoparticles of the ferrite group are of current research interest due to their wide range of industrial, biomedical and environmental applications.^3,16,17^ Cobalt–ferrite is an efficient member of the ferrite family with dominant properties such as chemical stability, high mechanical stability, wear resistance, high anisotropy and medium saturation magnetization. Diverse applications of cobalt–ferrite have been obtained by proper surface modification.^18^ Some examples of functionalized cobalt–ferrite NPs (CFNPs) include alginate-coated CFNPs (carrier for hyperthermia and targeted delivery),^19^ ethanolamine-functionalized CFNPs (immobilizer of cellulase enzyme *via* carbodiimide cross-link chemistry),^3^ polyvinyl alcohol-functionalized CFNPs and Au-coated CFNPs (for biomedical applications),^4,20^ and Rh-supported CFNPs (for catalytic activity towards hydroformylation reaction of olefins).^21^ Besides biomedical and catalytic applications, some magnetic cobalt–iron oxide NPs have also demonstrated potential in the removal of pollutants; for instance, biotin- and lawsone-coated CFNPs have been utilized to remove Rhodamine dye and lead from aqueous systems.^22^ CoFe_2_O_4_ with many other ferrites (MnFe_2_O_4_, MgFe_2_O_4_, ZnFe_2_O_4_, CuFe_2_O_4_, NiFe_2_O_4_ and CoFe_2_O_4_) has been utilized by Hu *et al.* (2007) in the removal of Cr(vi).^17^ PEG-coated CoFe_2_O_4_ NPs have shown selective removal of Congo Red compared to Methyl Orange and Methyl Blue.^18^ Doping with trivalent metal ions (Ni^3+^, Gd^3+^ and other rare-earth metals) has been found to be effective in enhancing the adsorption capacity and surface properties of cobalt–ferrite NPs.^18,23^ A report by Casbeer *et al.* (2012) reviews the photocatalytic activity of various metal-ferrites including CoFe_2_O_4_ alone or with other metal composites for the degradation of various dyes.^24^ Although the literature shows the verified role of different amine-functionalized cobalt–iron oxide NPs for various biomedical uses,^3,19^ their role in the removal of toxic azo dyes has not yet been fully established as the data in this field is very limited. A study done using ethanolamine-functionalized CFNPs has been retrieved in this regard that indicates the potential of these CFNPs in the adsorptive removal of three anionic azo dyes,^25^ showing the scope of further study of other amine-functionalized CFNPs in dye removal. Furthermore, no comparative dye removal study using different amine-functionalized cobalt–iron oxide NPs against different azo dyes has been reported so far. Such a study will be important to evaluate the structural effects of various azo dyes on their removal by different amine-functionalized adsorbents and to provide selective use of amine adsorbents for future removal of certain azo dyes. To the best of our knowledge, CFNPs functionalized with hydrazine and dodecylamine have not been used yet in a single comparative study against a variety of structurally different anionic azo dyes.

Therefore, the current study is aimed at synthesizing and characterizing two types of cobalt–iron oxide magnetic nanoparticles functionalized with hydrazine (CoFeNPs1) and dodecyl amine (CoFeNPs2) using the chemical coprecipitation method. The relative ability of these amine-functionalized NPs to remove six structurally different anionic azo dyes from water solutions was investigated and compared considering different factors such as the size of the dye and CoFeNPs, contact time, and functional groups on the dye and CoFeNPs for structural optimization and selectivity in dye removal. Electronic and infrared spectral studies and various kinetics and isotherm models were applied with desorption analysis to gain further insight into the mechanism of dye removal by amine-functionalized CoFeNPs.

## Experimental

### Materials

**Fig. 1:**
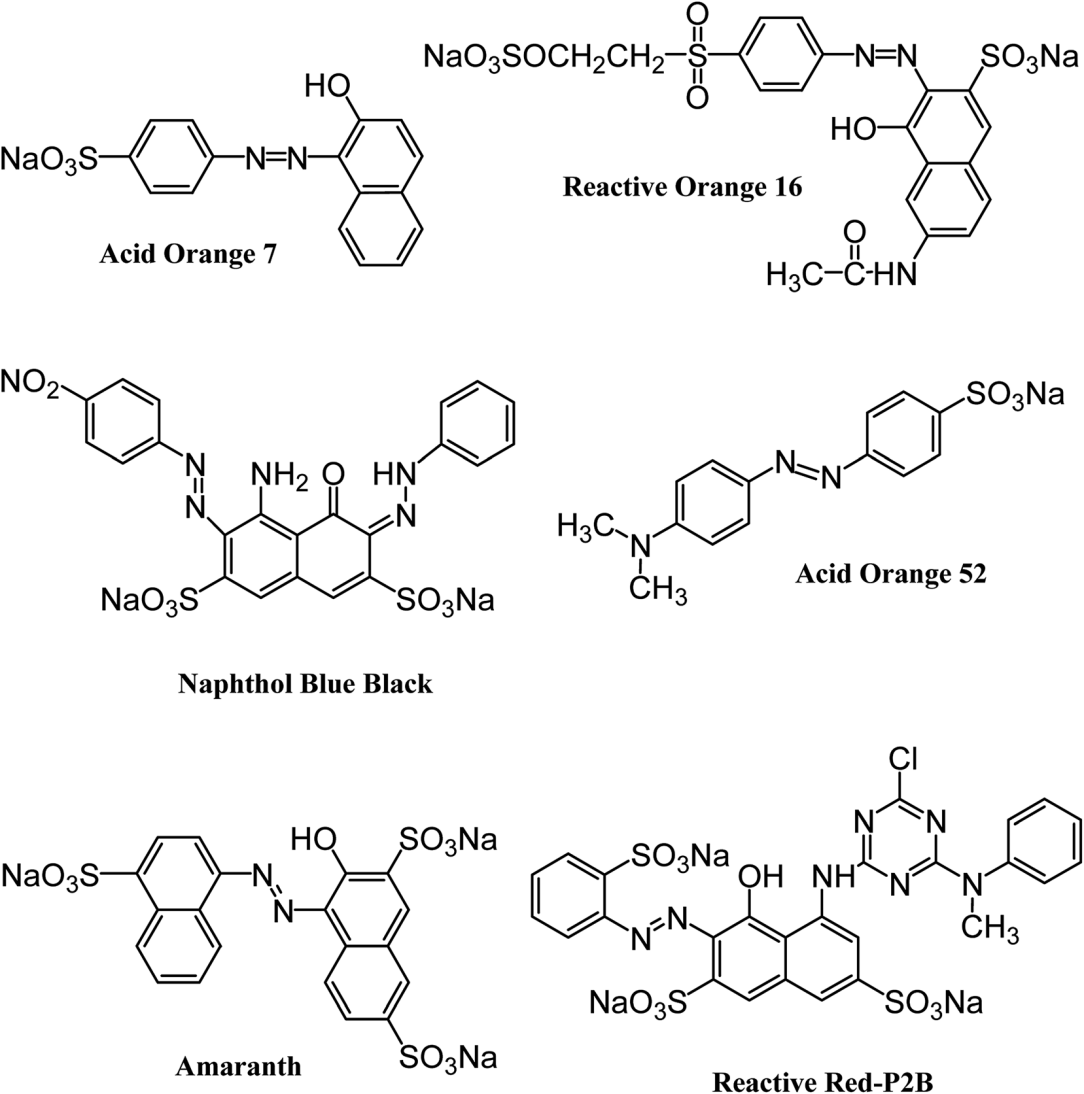
Structure of six different anionic azo dyes used in the current study.

All the reagents and chemicals applied in the present study were of analytical grade. They were utilized with no additional purification. Iron and cobalt metal salts (CoCl_2_·6H_2_O, FeCl_3_·6H_2_O) and dodecylamine (CH_3_(CH_2_)_11_NH_2_) were procured from Merck (Germany). Hydrazine monohydrate (98+%) was purchased from Alfa Aesar (England). Naphthol Blue Black, Acid Orange 52 and Acid Orange 7 dyes were acquired from Sigma-Aldrich (Germany). Reactive Orange 16 and Amaranth dyes were supplied by AVONCHEM (UK) and BDH Laboratory Supplies (UK), respectively. Commercial Reactive Red P2B was provided by Oh-Young Company (Korea). The structures and important characteristics of the selected dyes are provided in Fig. 1 and Table 1, respectively. Distilled water was deionized prior to preparing the required solutions through an ELGA Cartridge (Type C114).

**Table 1:** Dyes used in the current study and their properties

S. no.	Dye name and symbol	Molecular formula	Molar mass (g mol^−1^)	*λ*_max_ (nm)
1	Acid Orange 52 (AO52)	C_14_H_14_N_3_NaO_3_	327.33	459
2	Acid Orange 7 (AO7)	C_16_H_11_N_2_NaO_4_S	350.32	483
3	Amaranth (AMR)	C_20_H_11_N_2_Na_3_O_10_S_3_	604.47	519
4	Naphthol Blue Black (NBB)	C_22_H_14_N_6_Na_2_O_9_S_2_	616.48	615
5	Reactive Orange 16 (RO16)	C_20_H_17_N_3_Na_2_O_11_S_3_	617.54	490
6	Reactive Red-P2B (RR-P2B)	C_26_H_18_N_7_Na_3_O_10_S_3_	788.07	543

### Synthesis of amine-functionalized magnetic nanoparticles

**Scheme 1:**
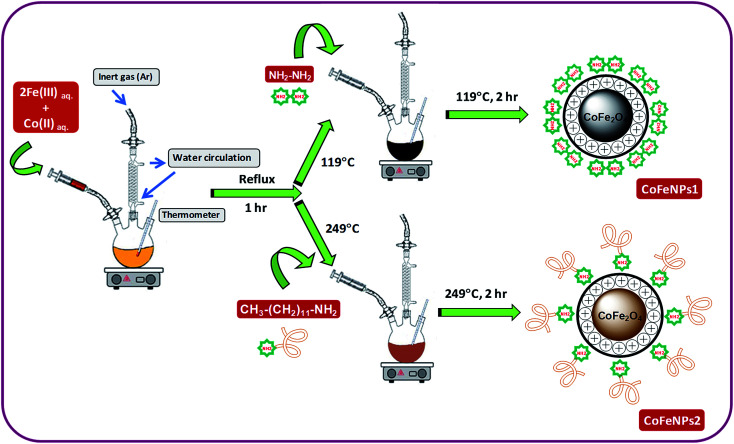
Synthesis of cobalt–iron oxide NPs (CoFeNPs) functionalized with hydrazine (CoFeNPs1) and dodecylamine (CoFeNPs2).

Two types of magnetic cobalt–iron oxide nanoparticles (CoFeNPs1 and CoFeNPs2) functionalized with two different amine reagents, hydrazine and dodecylamine, respectively, were synthesized using a simple and economic one-step coprecipitation method in aqueous medium.^26^Scheme 1 shows the synthesis of CoFeNPs1 and CoFeNPs2, and possible modes of bonding of functionalized amines to CoFe_2_O_4_ in the resulting CoFeNPs.

#### Synthesis of type 1 cobalt–ferrite nanoparticles (CoFeNPs1)

Initially, FeCl_3_·6H_2_O (5.40 g) and CoCl_2_·6H_2_O (2.38 g) were separately dissolved in 15 mL of deoxygenated distilled-deionized water and mixed. The solution mixture containing metal salts was constantly stirred at 400 rpm with heating at first to 70 °C for 20 min and then the temperature was gradually increased up to boiling for 1 h under reflux using an MS-H280-PRO digital hot plate (SCILOGEX). After 1 h of stirring, the temperature on the hot plate was adjusted to 119 °C (*i.e.*, boiling point of hydrazine hydrate), and 10.5 mL of hydrazine hydrate (20 M) was abruptly added and continuously stirred for 2 h. Throughout the reaction, the solution was continuously bubbled with argon gas to prevent possible oxidation of Co(ii) with air.^27^ The resulting black precipitates of magnetic cobalt–ferrite nanoparticles (CoFeNPs1) were detached from the mixture by an applied magnetic flux. The separated black solid was washed many times with water and ethanol until it was free from chloride ions and then with hexane and ultimately dried at room temperature for 24 h under vacuum.

#### Synthesis of type 2 cobalt–ferrite nanoparticles (CoFeNPs2)

CoFeNPs2 were prepared by following almost the same procedure as mentioned for CoFeNPs1 except for the surface functionalizing amino agent; dodecylamine was used in place of hydrazine to prepare CoFeNPs2. In brief, FeCl_3_·6H_2_O and CoCl_2_·6H_2_O were dissolved in deionized water and mixed in 2 : 1 molar ratio under an inert atmosphere. The mixture was constantly stirred at fixed velocity (400 rpm) and heated gradually up to boiling under reflux for 1 h. Afterward, 23.5 mL of dodecylamine (4.305 M) was quickly added at a temperature of 249 °C (B.P. dodecylamine) and stirred further for 2 h under an inert (argon) atmosphere. The resulting burnt brown precipitate of cobalt–ferrite nanoparticles (CoFeNPs2) was removed from the mixture using an external magnet, washed with water, ethanol and hexane sequentially and subsequently dried under vacuum at room temperature for 24 h.

### Characterization of CoFeNPs1 and CoFeNPs2

The surface functional groups and bonding modes in the CoFeNPs were confirmed from infrared spectra recorded using an FT-IR (Fourier-transform infrared) spectrometer (IR-460, Shimadzu) at 400–4000 cm^−1^ as KBr pellets. The apparent morphology, dimensions and composition/purity of the synthesized CoFeNPs were determined using a scanning electron microscope (JSM-6380A model, JEOL, Japan) containing C-coated Cu grids (voltage 20 kV) equipped with an EDS (energy-dispersive X-ray spectroscopy) detector (model EX-54175jMU, Jeol, Japan). For EDS analysis, the sample was enclosed with a 300 Å gold film. An atomic force microscope (model Agilent 5500) run in tapping form was also used for morphological analysis. To check and compare the thermal stability of CoFeNPs1 and CoFeNPs2, each sample (130 mg) was initially heated in an oven (Heraeus T 5028, Germany) from 25–250 °C and then in a muffle furnace (Thermolyne™ FB1310M) from 300–700 °C under oxidative conditions. To get the weight of the samples at different temperature points in the tested range, the samples were heated to preset temperature points until constant weights were observed. The zeta potential (surface charge) of both CoFeNPs was measured for comparison using a Malvern (UK) Zetasizer Nano ZS90 instrument taking 0.5 g L^−1^ samples suspended in deionized water at various pH values (1–14).

### Dye removal experiments

**Fig. 2:**
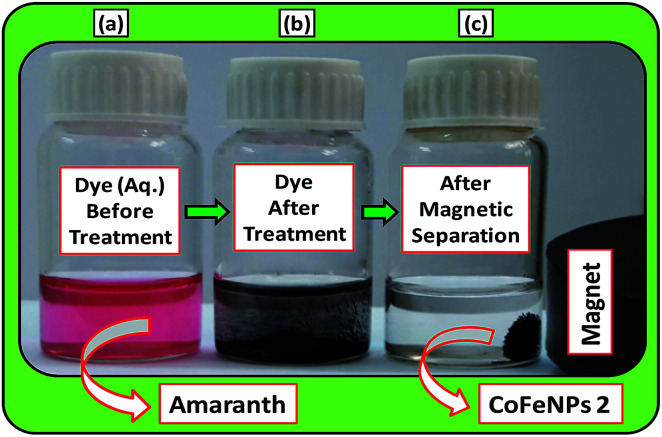
A real photograph showing the use of the magnetic cobalt–iron oxide nanoparticles (CoFeNPs2) used in our study as an adsorbent to remove Amaranth dye. (a) Aqueous dye solution before adsorption, (b) dye after adsorptive treatment and (c) magnetic separation after treatment.

Batch-mode adsorption studies were performed using amine-functionalized cobalt–iron oxide nanoparticles (CoFeNPs1 and CoFeNPs2) as adsorbents to remove six structurally different anionic azo dyes (Table 1, Fig. 1) from their aqueous solutions: Acid Orange 52 (AO52), Acid Orange 7 (AO7), Amaranth (AMR), Naphthol Blue Black (NBB), Reactive Orange 16 (RO16) and Reactive Red-P2B (RR-P2B), and the adsorption efficiency of both CoFeNPs was compared. For this purpose, an aqueous solution of each dye (15 mL) under fixed and similar conditions of dye concentration (0.02 mmol L^−1^), temperature (30 °C) and pH (6) was exposed to a specified dried mass of CoFeNPs (0.67 g L^−1^). The reaction mixture was stirred in a shaking thermostat water bath (SWB-A, BIOBASE) at 130 rpm until no further removal of dye took place or an equilibrium was established. This was followed by separating the dye-loaded CoFeNPs from the residual dye solution using a simple magnet (Nd–Fe–B magnet disk) as shown in some real images presented in Fig. 2 for the removal of amaranth dye by CoFeNPs2. A Shimadzu UV-240 (Hitachi U-3200) UV-visible spectrophotometer was applied to analyze the change in the absorbance of the dye. The absorbance of the dye solution was observed at dye *λ*_max_ and dye concentration was obtained using the calibration curve of the dye to determine the percent dye removal and adsorption capacity (*q*) of the CoFeNPs. The overlaid UV-visible spectra of six dyes at the initial concentration (0.02 mmol L^−1^) under the specified conditions without adsorbent, used as a reference or control, are provided in Fig. S1†. The efficiency of dye removal (% adsorption) and the extent of dye adhered onto the CoFeNPs (*q*, mg g^−1^) at various shaking time intervals were calculated using the following formulae:
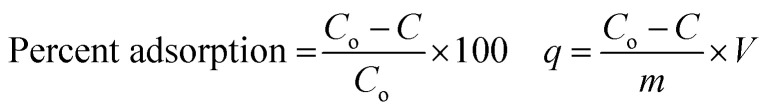
where *C*_o_ and *C* represent the initial dye concentration in the solution (mg L^−1^) and the dye concentration in the supernatant (mg L^−1^), respectively. *V* and *m* correspond to the volume of dye solution (L) and dry mass of the amine-CoFeNPs (g), respectively. In the above equations, *C* replaces *C*_e_ and *q* replaces *q*_e_ for equilibrium data, and hence *C*_e_ and *q*_e_ denote equilibrium dye concentration in the liquid (mg L^−1^) and equilibrium adsorption capacity of amine-CoFeNPs (mg g^−1^), correspondingly.

Each experiment was performed in triplicate, and their mean values under ±5% maximum deviation were considered for data analysis. To demonstrate the dye adsorption behavior and determine the adsorption mechanism by both amine-CoFeNPs with potential rate-governing steps, five different models of kinetics (pseudo-first order, pseudo-second order, Elovich, intra-particle diffusion and Boyd) were applied to the experimental data (collected at various time intervals) of removal of all dyes using the linear regression tool in Microsoft Office (Excel 2007 solver). RO16 was selected as a model anionic azo dye for further studies on pH effect (2–12), equilibrium adsorption isotherms, *i.e.*, Langmuir and Freundlich (12.4–49.4 mg L^−1^ dye at pH 4), thermodynamics (30–90 °C) and adsorbent reusability (eluents: 1 M HCl, 2 M NaOH, MeOH, MeOH/CH_3_COOH 9 : 1 v/v mixture) for dye removal by both amine-CoFeNPs. The other experimental conditions for the studies of the isotherms, kinetics, pH effect, thermodynamics and reusability are the same as mentioned initially for the preliminary adsorption assessments.

## Results and discussion

### Characterization of CoFeNPs1 and CoFeNPs2

The magnetic CoFeNPs1 and CoFeNPs2 were prepared by green, facile and inexpensive coprecipitation of metal salts under aqueous conditions (Scheme 1), and their identity was confirmed by FT-IR spectroscopy, SEM-EDS, AFM and oxidative thermal degradation studies.

#### FT-IR spectroscopy

**Fig. 3:**
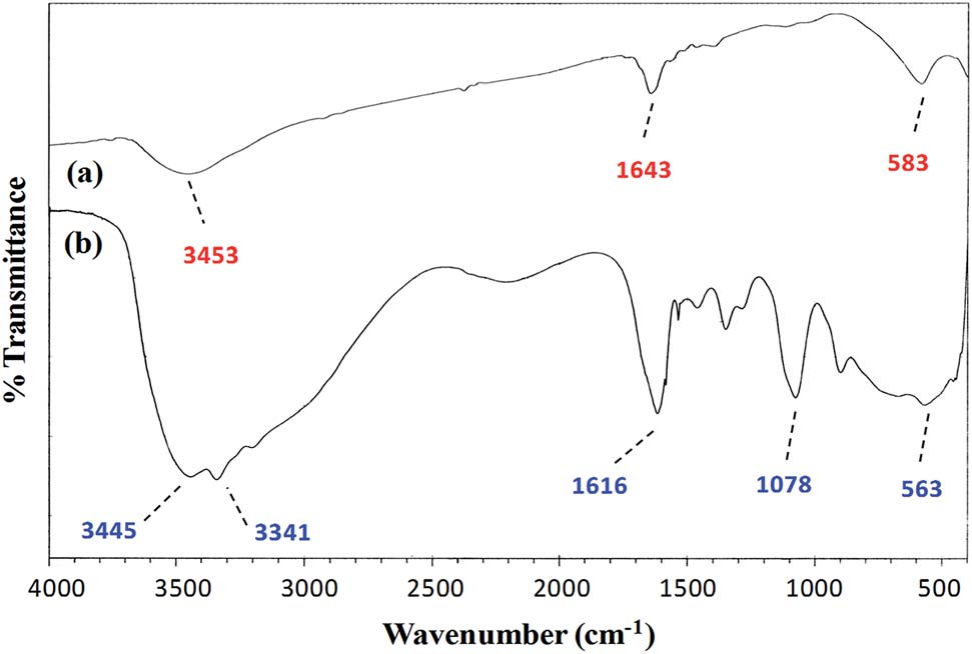
FT-IR spectrum of (a) hydrazine-functionalized cobalt–iron oxide NPs (CoFeNPs1) and (b) hydrazine hydrate.

**Fig. 4:**
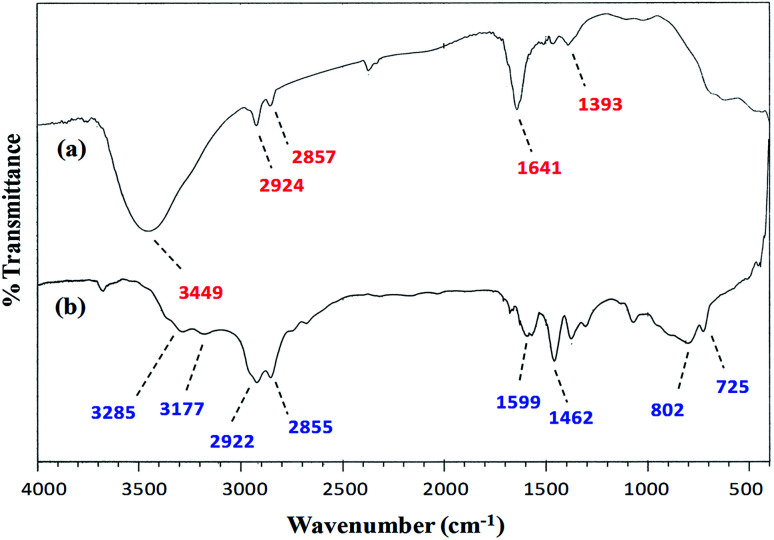
FT-IR spectra of (a) dodecylamine-functionalized cobalt–iron oxide NPs (CoFeNPs2) and (b) dodecylamine.

A comparison of the FT-IR (vibrational) spectra of each of the synthesized cobalt–iron oxide nanoparticles (CoFeNPs1 and CoFeNPs2) with those of the parent free amines (hydrazine and dodecylamine) (Fig. 3 and 4) successfully confirmed the surface functionalization or anchoring of cobalt–iron oxide nanoparticles with the respective amines. A sharp peak at 583 cm^−1^ in the vibrational spectrum of CoFeNPs1 (Fig. 3a) and a low intensity peak around the same region in the vibrational spectrum of CoFeNPs2 (Fig. 4a) correspond to intrinsic Fe^3+^–O^2−^ vibrations in spinel cobalt–ferrite.^25,26^ The lower intensity may be due to less exposed M–O bonds surrounded by adsorbed large dodecylamine molecules in CoFeNPs2. The primary amine stretchings (symmetric and asymmetric) in hydrazine hydrate are revealed by a pair of peaks at 3341 and 3445 cm^−1^ (Fig. 3b).^28^ These vibrations are shifted to 3453 cm^−1^ as a low intensity single band after bonding of the amine group of hydrazine to NPs in CoFeNPs1 (Fig. 3a). This band is a result of overlapping of peaks of OH and NH stretchings.^29^ Likewise, the amine stretching peaks of dodecyl amine, appearing at 3177 and 3285 cm^−1^ (Fig. 4b), were also shifted to 3449 cm^−1^ after anchoring of dodecylamine to the NPs surface in CoFeNPs2 (Fig. 4a). The alkyl chain C–H stretching peaks of dodecylamine at 2922 and 2855 cm^−1^ (Fig. 4b) are also explicitly visible in the FT-IR spectrum of CoFeNPs2 at 2924 and 2857 cm^−1^.^3,20^ The additional band at 1393 cm^−1^ for CoFeNPs2 is attributed to C–C stretching resonance. Hence, the appearance of certain peaks in the infra-red spectra of CoFeNPs1 and CoFeNPs2 confirms the attachment of hydrazine hydrate and dodecylamine, respectively, to the NPs surface.

**Fig. 5:**
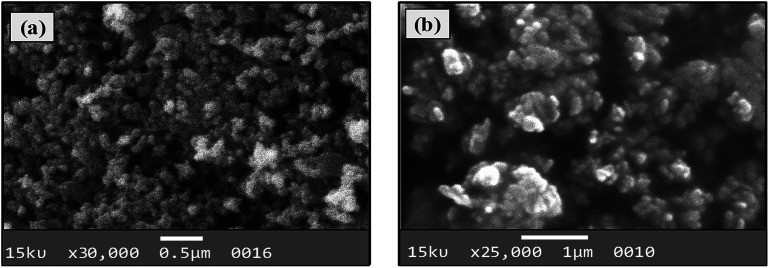
SEM images of (a) CoFeNPs1 and (b) CoFeNPs2.

#### Size, shape and composition

The morphology, size and composition of magnetic NPs significantly affect their chemical and physical characteristics. All of these properties in turn determine suitability for certain applications and depend on the ionic strength of the medium, pH value, reaction temperature, Fe^2+^/Fe^3+^ ratio and nature of the salts utilized (nitrates, sulfates, chlorides, *etc.*).^30^ The elemental composition and morphology of CoFeNPs1 and CoFeNPs2 were determined and compared using scanning electron microscopy equipped with energy-dispersive X-ray spectroscopy (SEM-EDS). The photomicrographs from the SEM study of the two CoFeNPs are shown in Fig. 5a and b.

**Fig. 6:**
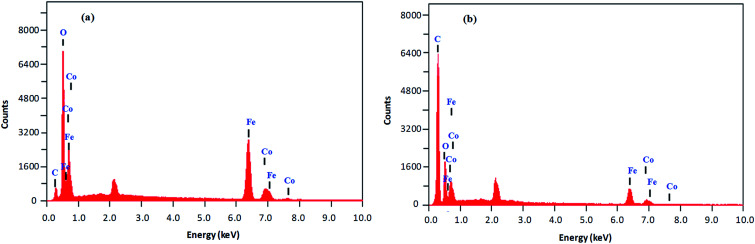
SEM-EDS analysis of (a) CoFeNPs1 and (b) CoFeNPs2.

Both types of amine-functionalized CoFeNPs revealed nanocrystal clusters of spherical shape. This agglomeration results from magnetic forces between the CoFeNPs.^31^ The SEM recorded size (diameter) for ten randomly selected isolated CoFeNPs1 and CoFeNPs2 ranged from 80–87 nm (avg. diameter = 84 nm) and 96–98 nm (avg. diameter = 97 nm), respectively. It is important to describe here that smaller particles (with low contrast seen) in the SEM images, particularly in the case of CoFeNPs1, did not enable measurements of diameter, so there is likely an error in the lower value of the given size range. The relatively larger cluster size of CoFeNPs2 may be due to increased agglomeration or larger size of the surface functionalizing material, *i.e.*, dodecylamine. A reduced agglomeration in CoFeNPs1 indicates better stabilization of the cobalt–iron oxide NPs with hydrazine compared to that provided by dodecylamine.^32^ The SEM-EDS spectra in Fig. 6 show the components of CoFeNPs1 and CoFeNPs2 with relative elemental counts. The elemental mass percentages from EDS analysis were found to be 20.37%, Co; 49.11%, Fe; 28.74%, O; and 1.78%, C for CoFeNPs1, whereas for CoFeNPs2 the elemental mass contents were 15.23%, Co; 39.08%, Fe; 20.96%, O; and 24.74%, C. The small carbon (C) content observed for CoFeNPs1 is because of the C-coated grids utilized in EDS analysis,^33^ while the significantly intense carbon peak observed for CoFeNPs2 confirms its surface functionalization with dodecylamine. The EDS analysis of CoFeNPs1 and CoFeNPs2 shows high purity of these compounds with cobalt–iron oxide stoichiometric composition as cobalt–ferrite (CoFe_2_O_4_).

**Fig. 7:**
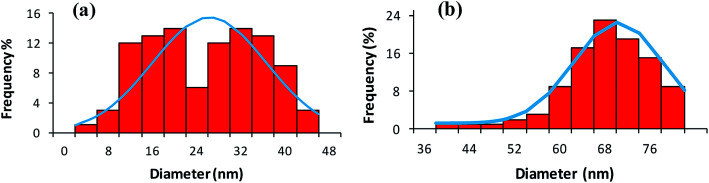
Size distribution histograms from AFM analysis of (a) CoFeNPs1 and (b) CoFeNPs2.

Atomic force microscopy (AFM) in tapping mode was also applied to confirm the relative size and morphological variations in CoFeNPs1 and CoFeNPs2. Tapping AFM provides better perception of the roughness of the surface with fine details of grain boundaries.^34^Fig. 7(a and b) and S2(a–d)† illustrate well resolved 2D and 3D topographic AFM images, and size distribution histograms for CoFeNPs1 and CoFeNPs2. AFM analysis also reveals polydisperse agglomerated isolated CoFeNPs of almost spherical morphology. The grain diameter size of CoFeNPs1 (4–44 nm, avg. diameter = 24 nm, Fig. 7a) is lower than the size of CoFeNPs2 (40–80 nm, avg. diameter = 68 nm, Fig. 7b). Thus, the results of AFM analysis (shape and relative size differences) for CoFeNPs1 and CoFeNPs are in agreement with the SEM outcomes. However, the relatively larger sizes of both CoFeNPs nanocrystal clusters from SEM compared to AFM may be due to the reason that the two instruments are not cross-calibrated, and they measure particle dimensions under different principles or criteria. The difference in the sampling of sub-populations (dispersion levels) for the two methods may also count. AFM measures the diameter of spherical particles using height dimensions (*z*-axis data) with high resolution, while SEM measures lateral dimensions (*x*- and *y*-axis data) requiring lateral magnification for optimized resolution. With agglomerated but smaller nanocrystal clustered samples of CoFeNPs, AFM analysis seems more accurate and precise with greater resolution for their size measurement.^35^

#### Thermal degradation studies

**Fig. 8:**
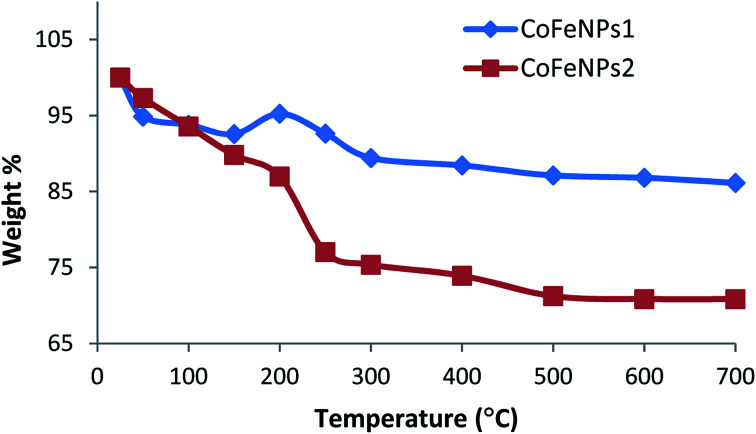
Variation of mass (weight%) of CoFeNPs1 and CoFeNPs2 with respect to temperature change under aerobic conditions.

To confirm the thermal stability, surface functionalization and thermal degradation behavior of the synthesized CoFeNPs1 and CoFeNPs2, these particles were thermally treated from 25 to 700 °C in an identical manner under aerobic conditions. At certain temperature intervals, the powder mass of both CoFeNPs was manually checked and their weight% is plotted *versus* temperature as shown in Fig. 8. CoFeNPs1 demonstrated significant thermal stability shown by a small total mass loss of 13.87% in the tested temperature range, with the significant mass deduction (about 76.28% of the total weight loss) before 300 °C. In contrast, about 2 times higher net mass loss (29.16%) was observed for CoFeNPs2 up to 700 °C, indicating higher mass coating of thermally degradable amine in CoFeNPs2. Fig. 8 illustrates that the two types of CoFeNPs show thermal degradation in at least three well-resolved stages.

The first step of weight loss up to 150 °C is assigned to libration of water molecules from the CoFeNPs surface, where an initial mass loss of about 6% up to 100 °C is attributed to physisorbed water, while further loss in weight (7–10%) up to 150 °C corresponds to chemisorbed water.^15^

An apparent unexpected mass increase of about 3% from 150 to 200 °C observed for CoFeNPs1 could be due to the adsorption of oxygen on the nanoparticle surface, which may penetrate into the core of the nanoparticles under aerobic conditions. The subsequent 5.8% mass loss up to 300 °C for CoFeNPs1 is probably due to the release of gases (N_2_, NH_3_, H_2,_*etc.*) from the surface, owing to the coated hydrazine.^36^ Compared to the small weight loss of only 2.6% for CoFeNPs1 at 200–250 °C, CoFeNPs2 shows a sharp decline in mass of about 10% (yielding 22.96% net weight loss) within the same temperature range, coinciding with the boiling point range of the coated dodecylamine, *i.e.*, 247–249 °C. This indicates the release or instigation of decomposition of the coated dodecylamine available on the CoFeNPs2 surface. The last stage of thermal degradation for CoFeNPs2 from 250–500 °C, comprising about 6% weight loss, mainly corresponds to the release of CO_2_ along with coke formation due to the presence of the long alkyl chain in the coated dodecylamine. The coke formation is confirmed by a pronounced color change of the CoFeNPs2 from burnt brown to black brown, and then finally turning into a fully black powdery residue on raising the temperature from 25 to 250 °C and then to 700 °C, respectively (Fig. S3†). Unlike CoFeNPs2, CoFeNPs1 showed a slight color change on heating, turning from black to blackish grey with a shinier granular texture after 250 °C (Fig. S3†).

The slight color modification and smaller weight loss of CoFeNPs1 compared to the more intense color change and drastic weight loss of CoFeNPs2 with temperature change indicates better thermal stability of CoFeNPs1 compared to CoFeNPs2. The difference in thermal stability of the two CoFeNPs can be associated with morphological differences in the individual particles. Coating of cobalt–ferrite NPs with hydrazine (CoFeNPs1) gives well-defined monocrystalline nanoclusters (smaller), while surface coating of cobalt–ferrite with dodecylamine (CoFeNPs2) leads to the growth of rather polycrystalline nanoclusters (larger), as evident from the AFM and SEM studies. Due to the presence of grain boundaries, polycrystallinity may significantly influence the susceptibility/stability of the oxide.^36^

### Dye removal studies

#### Screening of anionic azo dyes for adsorption onto amine-functionalized CoFeNPs

**Fig. 9:**
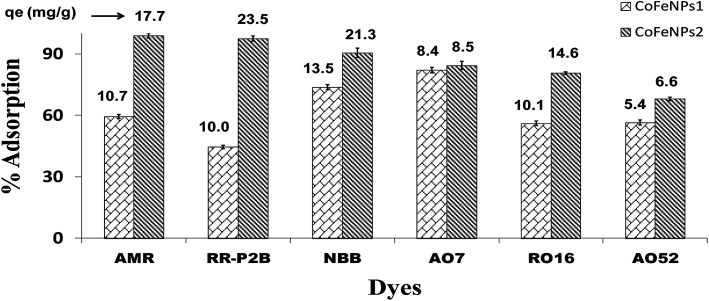
Comparison of the adsorption ability of CoFeNPs1 and CoFeNPs2 for six anionic azo dyes (AMR, RR-P2B, NBB, AO7, RO16 and AO52) [conditions: initial dye concentration 0.02 mmol L^−1^, temperature 30 °C, pH 6, and adsorbent dose 0.67 g L^−1^].

Six different anionic dyes, NBB, RO16, AO7, AMR, RR-P2B and AO52 (Table 1, Fig. 1) were screened for their potential to be removed by CoFeNPs1 and CoFeNPs2 from their aqueous solutions identically at 30 °C, pH 6, 0.02 mmol L^−1^ dye, and 0.67 g L^−1^ adsorbent (CoFeNPs1 or CoFeNPs2) dosage. Fig. 9 shows the comparative equilibrium percent removal and adsorption capacity of CoFeNPs1 and CoFeNPs2 for the six tested dyes. The dye removal efficiency of CoFeNPs1 against various dyes was found to be in the order of AO7 > NBB > AMR > AO52 > RO16 > RR-P2B. The trend of dye removal by CoFeNPs2 was different and observed as follows: AMR > RR-P2B > NBB > AO7 > RO16 > MO. The equilibrium of adsorption by CoFeNPs was fast and attained within 28–162 min.

Table 2 compares the removal efficiency of our synthesized CoFeNPs for each selected dye with reported adsorbents, photocatalysts or oxidants. No previous study was found with cobalt–ferrite NPs for the removal of AMR, RR-P2B or AO7. Previous data pertinent to the removal of NBB and RO16 by cobalt–ferrite is also scarce, and it focuses merely on dye catalytic degradation rather than adsorption.^24^ However, AO52 removal by cobalt–ferrite as an adsorbent and photocatalyst has been adequately studied previously.^50–53^ The adsorption capacities (*q*_max_) shown in Table 2 indicate that our CoFeNPs as adsorbents are comparable or better even under unoptimized conditions than many other adsorbents, including magnetiteCTAB NPs^37^ and alumina–polystyrene^38^ for AMR removal, *G. persica* biomass^42^ and activated carbon^43^ for NBB removal, beech wood sawdust^45^ and pelic soil^46^ for AO7 removal, rice husk activated carbon^10^ and zeolite–magnetite composite^49^ for RO16 removal, and CoFe_2_O_4_-reduced graphene oxide composites^51^ for AO52 removal. Considering the % dye removal data in Table 2, our CoFeNPs adsorbents can also provide a comparable or better adsorptive sequestration of the tested dyes from water than their degradative (photo/catalytic/oxidative) removal in many cases, for example, photooxidative removal by γ-ray/H_2_O_2_ for RR-P2B,^41^ photocatalytic removal by solar light/CeO_2_-carbon nanotubes for AO7,^48^ and oxidative-catalytic removal by H_2_O_2_/CoFe_2_O_4_ for NBB and RO16.^24^ Although many other adsorbents such as amine–PIM-1,^40^ CTAB–flax shives,^6^ CuFe_2_O_4_/activated carbon^47^ and CoFe_2_O_4_/MgAl-LDO,^50^ and some UV active photocatalysts such as CoFe_2_O_4_/ZnO^52^ and CoFe_2_O_4_–Fe_3_O_4_ (ref. 53) remove selected dyes in greater amounts (higher *q* or % removal) compared to the amount of dye removed by our CoFeNPs, most of such adsorbents or photocatalysts are much slower with an equilibrium time ≥ 300 min that could be as high as 72 h for amine–PIM-1 (ref. 40) compared to CoFeNPs (equilibrium time 28–162 min). Furthermore, the adsorption capacity or % removal of the studied CoFeNPs for selected dyes could be enhanced significantly after applying optimized conditions (pH, temperature, electrolyte, dye concentration, adsorbent dose, *etc.*); as evidence, we have maximized the adsorption capacity of CoFeNPs1 and CoFeNPs2 for RO16 from 10 to 68 mg g^−1^ and from 15 to 74 mg g^−1^, respectively (optimized conditions: pH 4, 30 °C, 0.67 g L^−1^ adsorbent dose). Hence, our CoFeNPs are very efficient nanoadsorbents that can provide inexpensive and rapid removal of many noxious anionic azo dyes from their aqueous solutions with comparable or superior proficiency compared to many other dye removing agents, offering the additional advantage of easy magnetic separation over non-magnetic dye removing agents.

**Table 2:** Comparison of the removal efficiency of CoFeNPs for six selected dyes with other adsorbents/photocatalysts^a^

Dye	Adsorbent/photocatalyst	*q*_max_ or % removal^b^	Dye (mg L^−1^)	*T* (°C)	pH	Dose (g L^−1^)	Time (min)	Ref.
Amaranth	Fe_3_O_4_–CTAB NPs	1 mg g^−1^	—	25	6	0.6	5	37
	Al_2_O_3_–polystyrene	15 mg g^−1^	—	30	2	2.5	120	38
	Fe_3_O_4_–polymer-MWCNT	47 mg g^−1^	—	25	6.2	0.1	360	39
	Amine–PIM-1	135 mg g^−1^*	50	20	7	0.05	4320	40
	CoFeNPs1/CoFeNPs2	11/18 mg g^−1^*	12.1	30	6	0.67	53/115	This work
Red-P2B	γ-ray (Co-60)/H_2_O_2_ (3 mM)	83.4%	100	25	9	—	100	41
	CoFeNPs1/CoFeNPs2	44.5/97.5%*	15.8	30	6	0.67	40/150	This work
Naphthol Blue Black	Fe_3_O_4_–histidine	167 mg g^−1^	—	30	4	0.2	45	15
	*Gracilaria persica* mass	9 mg g^−1^*	10.4	25	2	1.1	55	42
	Activated C (scrap tires)	15 mg g^−1^	—	25	3	0.8	120	43
	CTAB–flax shives	181 mg g^−1^	—	30	2	1	600	6
	CoFe_2_O_4_ + H_2_O_2_ (no irrad.)	68%	50	30	6.6	25	1440	24
	CoFeNPs1/CoFeNPs2	14/21 mg g^−1^, 73.7/90.5%*	12.3	30	6	0.67	105/162	This work
Acid Orange 7	Canola stalks	25.1 mg g^−1^	—	25	2.5	7.5	720	44
	Beech wood sawdust	5 mg g^−1^	—	25	7	2	180	45
	Pelic soil	4 mg g^−1^	—	30	2	50	240	46
	CuFe_2_O_4_/activated C	392 mg g^−1^	—	25	5.2	2	1440	47
	CeO_2_/CNTs + solar light	66.58%	40	25	5	0.5	240	48
	CoFeNPs1/CoFeNPs2	8/9 mg g^−1^, 82.1/84.3%*	7	30	6	0.67	105/150	This work
Reactive Orange 16	Activated C (rice husk)	19 mg g^−1^	—	30	11	0.003	30	10
	Fish scale–char	106 mg g^−1^	—	30	7	1	1440	7
	Zeolite/Fe_3_O_4_ composite	1 mg g^−1^	—	25	7	10	420	49
	CoFe_2_O_4_ + H_2_O_2_ (no irrad.)	21%	50	30	6.6	25	1440	24
	CoFeNPs1/CoFeNPs2	68/74 mg g^−1^, 72.0/97.2%*	—	30	4	0.67	30/75	This work
Acid Orange 52	CoFe_2_O_4_/MgAl-LDO	1220 mg g^−1^	—	25	6	0.2	≥400	50
	CoFe_2_O_4_/rGO	54.9%*	3.3	25	6	0.25	30	51
	CoFe_2_O_4_/ZnO + UV	94%	50	25	7	30	300	52
	CoFe_2_O_4_–Fe_3_O_4_ + UV	93%	3.3	25	6	0.2	300	53
	CoFeNPs1/CoFeNPs2	5/7 mg g^−1^, 56.4/68.0%*	6.5	30	6	0.67	28/93	This work

a All the data with (*) represent equilibrium adsorptive removal under unoptimized conditions except our data for RO16 that is at optimized conditions.

b All the values with mg g^−1^ unit and % values with (*) indicate adsorptive removal, while % values without (*) indicate photo-removal or photo-catalytic removal of dye.

#### Structural parameters affecting the adsorption of anionic azoic dyes onto amine-CoFeNPs

There are a number of structure-related factors that are expected to affect the adsorption of amine-functionalized CoFeNPs for anionic azo dyes, and these are described below.

#### Effect of surface charge of adsorbent

**Fig. 10:**
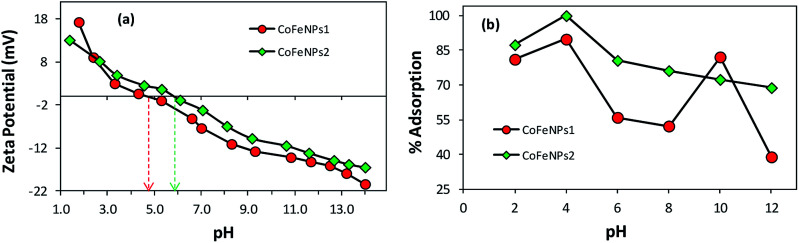
Comparative (a) zeta potential analysis of CoFeNPs1 and CoFeNPs2 and (b) pH effect on RO16 adsorption by CoFeNPs1 and CoFeNPs2 [conditions: initial RO16 concentration 0.02 mmol L^−1^, temperature 30 °C, and adsorbent dose 0.67 g L^−1^].

As evident from Fig. 9, the use of CoFeNPs2 significantly enhanced the removal of each dye compared to CoFeNPs1. This may be anticipated due to more positive or less negative charge on the CoFeNPs2 surface compared to that on CoFeNPs1 at the studied pH value (pH 6). Since all azo dyes utilized here are anionic due to the presence of sulfonate groups, they can better interact with more positively charged adsorbents. To confirm such effect of charge on dye adsorption and to determine the pH of zero point charge (pHzpc) for the two adsorbents, zeta potential (mV) measurements were performed at various pH values (Fig. 10a) in water. The pHzpc value for CoFeNPs1 and CoFeNPs2 was found to be around 4.7 and 5.8, respectively. The pHzpc of the adsorbents and their zeta potential values at pH 6 confirm that CoFeNPs1 is more negative compared to CoFeNPs2 at the studied pH, probably because of better neutralization of the ferrite positive charge by free amino electrons in CoFeNPs1 (two amino groups per hydrazine coated) compared to CoFeNPs2 (single amino group per dodecylamine coated) (Scheme 1), hence confirming the effect of CoFeNPs charge on the removal of negatively charged azoic dyes. Furthermore, this removal of anionic dyes can be enhanced by conducting adsorption studies at lower (more acidic) pH conditions, as confirmed by Fig. 10b showing maximum adsorption of RO16 at pH 4 by CoFeNPs1 (89.77%, *q*_e_ = 15.8 mg g^−1^) and CoFeNPs2 (99.9%, *q*_e_ = 17.60 mg g^−1^), due to increased positive charge on the NPs surface owing to cationic amines (–NH_3_^+^). However, further increase in pH could not increase dye removal due to dissolution of the CoFeNPs in strongly acidic medium. This interpretation is consistent with the study of Salazar-Rabago *et al.* (2017); they observed an increase in the adsorption capacity of an anionic adsorbent (natural sawdust) for a cationic dye (Methylene Blue) on increasing the pH value due to increased anionic charge on the adsorbent surface and hence increased electrostatic attraction between the adsorbent and dye.^54^ The significant uptake of anionic dyes by CoFeNPs at pH 6, in spite of there being some obvious electrostatic repulsion among the dyes and CoFeNPs, suggests that besides involving simple electrostatic attraction, other linkages, such as dispersive and van der Waals forces, may also contribute to the adsorption of negatively charged azo dyes onto the CoFeNPs surface.^55^ Additionally, CoFeNPs1 may also involve hydrophilic interactions in dye removal based on the enhanced adsorption of RO16 at pH 10 (Fig. 10b), as alkaline pH enhances the hydrophilicity of CoFeNPs1 (amine-rich); however, further alkalinity results in increased negative charge on the CoFeNPs causing strong repulsion between the NPs and anionic dyes and thus reducing dye adsorption.

#### Presence of hydrophobic groups on the adsorbent

The presence of a long alkyl chain in dodecylamine covering the CoFeNPs2 surface may provide a platform as a basal plane for hydrophobic interactions of CoFeNPs2 with hydrophobic groups (aromatic rings) in the dye molecules, which may also be responsible for the greater adsorption efficiency of CoFeNPs2, compared to the removal efficiency of CoFeNPs1 that does not exhibit such hydrophobic groups.^51^ The hydrophobicity difference among the two CoFeNPs was confirmed by their relative adsorption of hydrophobic dye Rose Bengal (RB). At predefined unoptimized experimental conditions, the RB adsorption by CoFeNPs1 and CoFeNPs2 was 64.7% (*q*_e_ = 11.2 mg g^−1^) and 99.9% (*q*_e_ = 28.5 mg g^−1^), respectively.

#### Particle size or surface area of the adsorbent

Usually, enhancement of the particle size of an adsorbent negatively affects the degree of adsorption of the adsorbent for an adsorbate due to decreased surface area as documented by Yean *et al.* (2005) after studying in detail the effect of size of magnetite particle on arsenite and arsenate adsorption.^56^ Contrary to this study, in our case, CoFeNPs2 with a relatively larger size is a more efficient adsorbent than smaller CoFeNPs1. Therefore, it can be suggested that size or surface area is not the only parameter that controls the extent of adsorption, and other factors such as hydrophobic character in the adsorbent may be more dominating in controlling the adsorption of anionic dyes.

#### Size, complexity and hydrophobic character in anionic dyes

AO52 and AO7 are the simplest or smallest while RR-P2B and AMR are the largest and most complicated dyes among the tested anionic azo dyes (Table 1, Fig. 1). CoFeNPs2 showed the lowest dye removal efficiency (67.98%) for AO52, while the highest for AMR (98.85%) and second highest for RR-P2B (97.46%). The reason may be that the larger dyes possess a larger number of carbon atoms (*e.g.*, 26 for RR-P2B and 20 for AMR) compared to the carbon atoms exhibited by smaller dyes (*e.g.*, 14 for AO52 and 16 for AO7), facilitating better hydrophobic interactions with the hydrophobic alkyl chain on CoFeNPs2 and hence imparting better removal of larger dyes by larger (size compatible) CoFeNPs2. In contrast, CoFeNPs1 revealed the lowest removal efficiency for the largest RR-P2B (44.47%) and the highest removal ability for small AO7 (82.08%). It is likely that increased steric hindrance due to larger dye size is responsible for the decreased dye removal ability of the smaller CoFeNPs1 against larger dyes. Furthermore, the hydrophobic interactions for CoFeNPs1 are not as important as for CoFeNPs2 due to the absence of surface hydrophobic functionalities in CoFeNPs1.

#### Number of sulfur atoms in the anionic dyes

Another interesting relationship observed was between dye removal ability and number of sulfur atoms. The dyes exhibiting a greater number of phenyl-sulfonate groups (*e.g.*, three in AMR and RR-P2B) resulted in better removal by CoFeNPs2 compared to dyes with a lower number of phenyl-sulfonate groups (*e.g.*, one in AO7 and AO52). Consistent with this finding, a recent study by Liu *et al.* (2019) also correlates the presence of sulfonyl groups and lower p*K*_a_ of dye molecules with higher adsorption capacities of magnetic Fe_3_O_4_/MIL-88A adsorbent for anionic dyes compared to cationic dyes lacking sulfonyl groups.^57^ A larger number of –SO_3_^−^ groups in the dyes renders greater negative charge, which can offer better electrostatic attraction with more positively- or less negatively-charged amine-functionalized adsorbents, favoring enhanced removal of more negative anionic dyes. Therefore, the possible mechanism of interaction between CoFeNPs2 and anionic dyes is suggested as electrostatic attraction and hydrophobic interactions. Unlike this, the adsorptive removal by CoFeNPs1 was negatively related to the number of sulfur atoms in the dyes molecules. This indicates that the electrostatic interactions between CoFeNPs1 and the dyes may not be significant and they interact with each other through a different mode of action, probably hydrophilic.

It can be inferred that amine-functionalized CoFeNPs could be selectively employed to remove different anionic azo dyes from textile effluents considering structural features such as size, complexity, charge and elemental composition (mainly number of S and C atoms) in the target dyes.

#### Contact time effect on the adsorption of amine-CoFeNPs

**Fig. 11:**
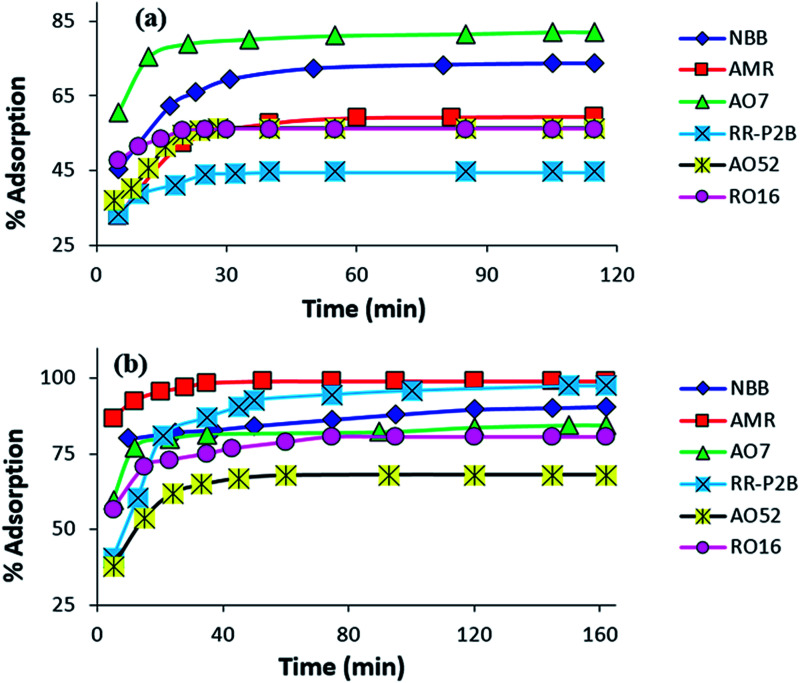
Influence of contact time on the adsorption of various anionic dyes (NBB, AMR, AO7, RR-P2B, AO52 and RO16) by (a) CoFeNPs1 and (b) CoFeNPs2 [conditions: initial dye concentration 0.02 mmol L^−1^, temperature 30 °C, pH 6, and adsorbent dose 0.67 g L^−1^].

The effect of stirring time on the removal efficiency of all six anionic dyes by CoFeNPs1 and CoFeNPs2 was analyzed, and the results are displayed in Fig. 11. The removal of each dye from its aqueous solution was increased with the contact time. The adsorption equilibrium was obtained in 28–115 min for CoFeNPs1 and 72–162 min for CoFeNPs2, indicating fast adsorption rates, which may be due to the nonporous nature of the adsorbent (amine-functionalized CoFeNPs), for which intra-particle diffusion is less dominant in slowing the adsorption rate.^8^ Fast adsorption of anionic azo dyes by these CoFeNPs is an important benefit of using such adsorbents at an industrial level.

### Mechanism of dye removal by amine-CoFeNPs (spectral studies)

#### Electronic spectra

**Fig. 12:**
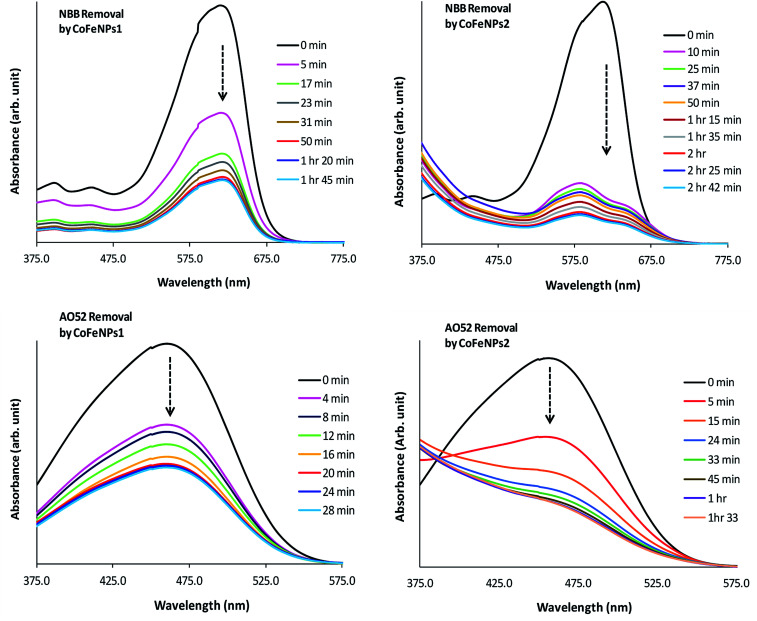
Comparative electronic spectra of the removal of NBB and AO52 at various time intervals by CoFeNPs1 (left) and CoFeNPs2 (right).

To gain a detailed insight into the mechanism of removal of various azo dyes by amine-functionalized CoFeNPs, the electronic spectra (375–800 nm) of the dyes at various time intervals during their removal by CoFeNPs1 and CofeNPs2 were collected and compared with each other (Fig. 12, S4 and S5†). Decrease in the absorbance confirms dye removal by the tested nanoadsorbents. The shape of the visible spectrum remains the same before and after adding CoFeNPs1 to any of the dye solutions, without affecting the *λ*_max_ or any new band appearing. This indicates that CoFeNPs1 interacts with each dye through a similar mechanism of action, that is adsorption, without any significant structural change or aggregation of the azo dye molecules.^58^

Unlike CoFeNPs1, CoFeNPs2 caused significant alteration in the shape of the intense chromophore (–N=N–) band in the visible spectrum of three dyes (AO7, NBB and AO52) with a shift in *λ*_max_ or giving new bands. This behavior was initially expected due to spontaneous degradation of the azo bond of these dyes by CoFeNPs2 under ordinary light and aerobic conditions, because NPs can produce some photocatalytic effect, induced by ordinary visible solar energy and dissolved oxygen in aqueous medium, causing dye degradation by generating OH˙ radicals.^24^ Additionally, this catalytic effect can be further associated with the presence of carbon in the ferrite structure as doping of a UV-active photocatalyst with carbon can make it visible light-active by reducing its band gap.^59^ However, extended studies of dye removal by CoFeNPs2 in the applied UV and visible light did not affect the dye removal ability of CoFeNPs2 for any dye, excluding the possibility of any photo-degradation (mainly of AO7, AO52 and NBB) by CoFeNPs2. A possible cause of the spectral shift in the dye absorption band by CoFeNPs2 may be the probable strong hydrophobic interaction of the azo dye rings with the alkyl chain of the coated surfactant, *i.e.*, dodecylamine, forming a hydrophobic azo dye–surfactant complex in the aqueous phase. Many azo dyes, such as Congo red, AO7, AO52 and 4-phenylazo-1-naphthylamine have also been shown previously to form dye–surfactant complexes (D_*m*_S_*n*_) in aqueous submicellar solutions.^60–62^ The stoichiometry of D_*m*_S_*n*_ complexes mainly depends on the surfactant alkyl chain length; C_8_–C_12_ surfactants give 1 : 1 complexes, while C_13_–C_18_ surfactants prefer 1 : 2 D_*m*_S_*n*_ associations.^63^ The D_*m*_S_*n*_ complexes/aggregates are well characterized by blue spectral shifts in *λ*_max_ and often an isosbestic point compared to the original dye chromophore band. In addition to strong hydrophobic interactions, these complexes may also involve ion-pair formation or hydrophilic interactions among polar groups of the surfactant and dye.^61,62^ The interaction of the β-nitrogen of the dye azo group with polar groups of the surfactant in strong dye–surfactant complexes may give rise to a new absorption band as observed in the spectrum of NBB dye around 575 nm.^63^ The change in the band shape or *λ*_max_ for the AO7, AO52 and NBB dyes by CoFeNPs2 may also be due to the formation of dye aggregates with CoFeNPs2. A red- or blue-shift in the dye absorption spectrum is a well-established characteristic of J-aggregates (head–tail, slipped stack arrangement), or H-aggregates (parallel plane-to plane stacking, sandwich-type arrangement) of dyes, respectively, in solutions, on NPs, or in the NPs assembly, owing to strong intermolecular attractive forces, *e.g.*, electrostatic and π–π interactions of dyes. The assembly of NPs and dye exhibits unique optical and electronic properties from dye aggregates and surface plasmon resonance from the NPs and offers chemical, biological and optical applications.^64,65^ Additional studies are necessary to confirm the actual cause of the spectral shifts for the AO7, AO52 and NBB dyes, whether involving dye–alkyl (D_*m*_S_*n*_) interactions or forming dye aggregates with NPs.

Since the absorbance for AO7, NBB and AO52 continued to decrease until equilibrium, the new species/aggregates may gradually adsorb onto CoFeNPs2 until equilibrium is established. Based on these results, the mechanism of removal of AO7, NBB and AO52 by CoFeNPs2 is suggested as dye aggregation and adsorption. The visible spectral change for three other dyes, RO16, RR-P2B and AMR, during removal by CoFeNPs2 is the same as that observed for CoFeNPs1, suggesting no degradation or aggregation of these dyes by CoFeNPs2, but removal by an adsorption phenomenon.^58^ This may be attributed to the less exposed hydrophobic benzene rings in the RO16, RR-P2B and AMR dyes, which are affluent with anionic sulfoxo groups (three groups), where dye adsorption by CoFeNPs2 through electrostatic attraction is more likely to be present.

#### Vibrational spectra

FT-IR spectroscopy was also successfully employed to confirm the predicted dye removal mechanism. Vibrational spectra of dye-treated CoFeNPs1 and CoFeNPs2 were compared with each other and also compared with the spectra of the respective control dyes. The FT-IR results for AO7 dye and treated CoFeNPs are provided in Fig. S6.†

The azo bond (–N=N–) stretching peak for AO7 dye appeared at 1562 cm^−1^ as depicted in Fig. S6c.†.^66^ The azo bond stretching is also clearly visible in the FT-IR spectrum of AO7-treated CoFeNPs1 at 1560 cm^−1^ (Fig. S6a†), showing adsorption of AO7 onto CoFeNPs1 without affecting its azo bond. The peaks at 1402 and 1119 cm^−1^ for AO7-treated CoFeNPs1 are also due to adsorbed AO7, as these peaks are also present in the infra-red spectrum of free AO7 at around the same positions (1400 and 1123 cm^−1^), associated with asymmetric and symmetric SO_2_ stretchings.^33^ Only slight shifting in the peaks of the sulfonate group after treatment proposes weak electrostatic attraction of the anionic sulfonate group with CoFeNPs1, most probably because of the positive ferrite core being properly covered by hydrazine and the weakly positive/more negative surface charge of CoFeNPs1 at pH 6 (Scheme S1†). The peak of O–H stretching of AO7 at 3449 cm^−1^ shifts significantly to 3474 cm^−1^ after adsorption of AO7 onto CoFeNPs1, probably because of strong hydrophilic interactions between AO7 and CoFeNPs1 through the dye OH group.

In contrast, the characteristic peak of the azo bond almost disappeared in the AO7-treated CoFeNPs2 spectrum (Fig. S6b†), probably because of polar interaction of the β-nitrogen of the AO7 azo group (with the coated surfactant in the dye–surfactant complex or within dye aggregated assemblies) adsorbed on CoFeNPs2. The other AO7 specific peaks in treated CoFeNPs2 were also absent or shifted significantly in the fingerprint region. The presence of some new peaks at 3420, 1625 and 1393 cm^−1^ for the AO7-treated CoFeNPs2 can be assigned to amine stretching, angular deformation of NH_2_, and SO_2_ group stretching, respectively, while the peak at 1026 cm^−1^ conforms to S=O stretching or C–H bending in the species adsorbed onto CoFeNPs2. Considerable shifting of the SO_2_ stretching peaks of AO7 after treatment with CoFeNPs2 highlights strong ionic interaction between the dye and CoFeNPs2. The reduction in the intensity of the pair of C–H stretching peaks (at 2855–2926 cm^−1^) of the untreated CoFeNPs2 (Fig. 5a) and free AO7 (Fig. S6c†) after treatment (Fig. S6b†) suggests hydrophobic interaction between the dye and CoFeNPs2 and the presence of adsorbed dye–surfactant complex or π–π stacked dye aggregates on the AO7-treated CoFeNPs2.

The peaks at 3416 and 3420 cm^−1^ for the AO7-treated CoFeNPs1 (Fig. S6a†) and CoFeNPs2 (Fig. S6b†), respectively, represent amine functionalities in the CoFeNPs adsorbents; however, these peaks are shifted significantly compared to that for native untreated CoFeNPs1 (3453 cm^−1^, Fig. 3a) and CoFeNPs2 (3449 cm^−1^, Fig. 4a), indicating strong interactions of the amino groups anchored on the CoFeNPs surface (mainly on CoFeNPs1) with AO7.^67^

Therefore, the mechanism of removal of AO7 by CoFeNPs1 is adsorption involving hydrophilic and weak ionic interactions, while that by CoFeNPs2 is dye aggregation and adsorption involving strong ionic and hydrophobic linkages. The FT-IR spectral results for the removal of other dyes by CoFeNPs1 and CoFeNPs2 were also consistent with the UV-visible spectral results and effects of structural factors, well validating the suggested mechanism of azo dye removal by the amine-functionalized CoFeNPs. The comparative mechanism of removal of azo dyes by CoFeNPs1 and CoFeNPs2 with all probable adsorbent–adsorbate interactions is proposed in Scheme S1† selecting AO7 and AMR, respectively, as these dyes provided the highest respective removal efficiencies.

### Dye removal kinetics

**Fig. 13:**
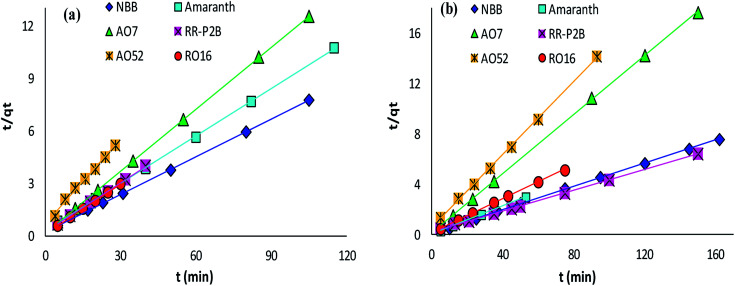
Pseudo-second order plots of the kinetics for the adsorption of anionic azo dyes onto (a) CoFeNPs1 and (b) CoFeNPs2.

**Fig. 14:**
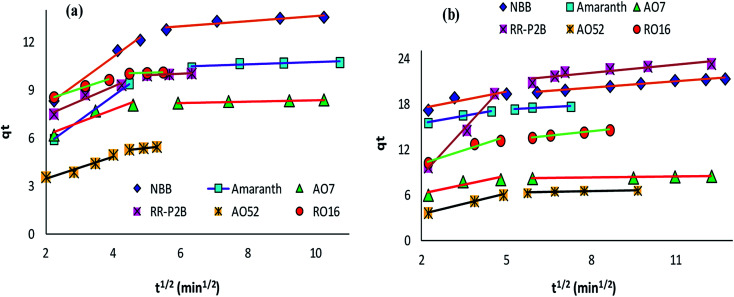
Intra-particle diffusion plots of the kinetics for the adsorption of anionic azo dyes onto (a) CoFeNPs1 and (b) CoFeNPs2.

**Fig. 15:**
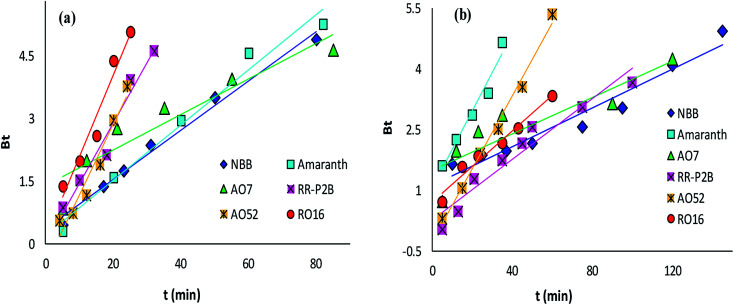
Boyd plots of the kinetics for the adsorption of anionic azo dyes onto (a) CoFeNPs1 and (b) CoFeNPs2.

The adsorption rate and potential rate-governing steps (chemical reaction processes, mass transport, *etc.*) can be evaluated by fitting the experimental data at various time intervals to appropriate kinetics models. Five different kinetics models have been examined for the adsorption of negatively charged azoic dyes onto CoFeNPs1 and CoFeNPs2: pseudo-first order, pseudo-second order, Elovich, intra-particle diffusion and Boyd (Fig. 13–15, S7 and S8†). The respective mathematical expressions and plotting parameters are given in Table 3 for comparison. The calculated kinetics parameters from each plot are shown in Table 4 for CoFeNPs1 and Table 5 for CoFeNPs2. Kinetics experiments were undertaken at initial dye concentration 0.02 mmol L^−1^, adsorbent dosage 0.67 g L^−1^, pH 6 and temperature 30 °C. Samples of different dyes were withdrawn at certain time intervals until the residual dye concentration became constant and were analyzed at *λ*_max_ of the dye for kinetics evaluation.

**Table 3:** Kinetics models applied and respective linear forms

Kinetics model	Linear equation	Plot
Lagergren's pseudo-first order	ln(*q*_e_ − *q*_*t*_) = ln *q*_e_ − *k*_1_*t*	ln(*q*_e_ − *q*_*t*_) *vs. t*
Pseudo-second order	*t*/*q*_*t*_ = 1/(*k*_2_*q*_e_^2^) + *t*/*q*_e_	*t*/*q*_*t*_*vs. t*
Elovich	*q*_*t*_ = (1/*b*)ln(*ab*)+ (1/*b*)ln *t*	*q*_*t*_ *vs.* ln *t*
Intra-particle diffusion	*q*_*t*_ = *k*_id_*t*^1/2^ + *C*_i_	*q*_*t*_ *vs. t* ^1/2^
Boyd model	*B*_*t*_ = −0.4977 − ln(1 − *F*)	*B*_*t*_ *vs. t*

**Table 4:** Kinetics parameters related to the adsorption of anionic azoic dyes onto CoFeNPs1^a^

Kinetics parameters	AO7	AO52	AMR	NBB	RO16	RR-P2B
**Experimental**						
*q*_e_	8.371	5.420	10.72	13.53	10.08	10.01

**Pseudo-first order model**						
*q*_e_ (mg g^−1^)	1.259	5.433	5.308	5.674	5.315	5.912
*k*_1_ (min^−1^)	0.043	0.167	0.066	0.059	0.196	0.143
*R* ^2^	0.8773	0.9475	0.9787	0.9886	0.9570	0.9659

**Pseudo-second order model**						
*q*_e_ (mg g^−1^)	8.482	6.227	11.11	14.03	10.54	10.59
*k*_2_ (g mg^−1^ min^−1^)	0.083	0.039	0.025	0.021	0.074	0.043
*R* ^2^	1	0.9936	0.9997	0.9999	0.9998	0.9996

**Elovich model**						
*a* (mg g^−1^ min^−1^)	5701	5.893	20.25	67.30	2328	109.8
*b* (g mg^−1^)	1.591	0.919	0.640	0.585	1.101	0.797
*R* ^2^	0.7802	0.9545	0.8971	0.9079	0.9749	0.9659

**Intraparticle diffusion model**						
*k*_id_ (mg g^−1^ min^−1/2^)	0.203	0.641	0.518	0.565	0.489	0.615
*C*_i_ (mg g^−1^)	6.612	2.204	6.106	8.659	7.614	6.479
*R* ^2^	0.5978	0.9648	0.7223	0.7299	0.9287	0.9008

**Boyd model**						
*R* ^2^	0.8773	0.9475	0.9787	0.9886	0.9570	0.9659

a Experimental conditions: pH = 6, dye concentration = 0.02 mmol L^−1^, temp. = 30 °C, adsorbent dose = 0.67 g L^−1^

**Table 5:** Kinetics parameters related to the adsorption of anionic azo dyes onto CoFeNPs2^a^

Kinetics parameters	AO7	AO52	AMR	NBB	RO16	RR-P2B
**Experimental**						
*q*_e_	8.495	6.585	17.66	21.34	14.57	23.49

**Pseudo-first order model**						
*q*_e_ (mg g^−1^)	1.152	5.272	3.723	4.227	4.357	10.95
*k*_1_ (min^−1^)	0.022	0.090	0.095	0.024	0.044	0.038
*R* ^2^	0.7871	0.9909	0.9644	0.9469	0.9702	0.9380

**Pseudo-second order model**						
*q*_e_ (mg g^−1^)	8.547	6.920	17.99	21.69	14.99	24.45
*k*_2_ (g mg^−1^ min^−1^)	0.068	0.035	0.058	0.013	0.023	0.006
*R* ^2^	0.9999	0.9994	0.9999	0.9996	0.9995	0.9993

**Elovich model**						
*a* (mg g^−1^ min^−1^)	1.19 × 10^4^	8.939	2.30 × 10^6^	2.11 × 10^7^	314.6	13.19
*b* (g mg^−1^)	1.713	0.946	1.061	1.036	0.654	0.024
*R* ^2^	0.7304	0.9150	0.9747	0.9521	0.9635	0.8940

**Intra-particle diffusion model**						
*k*_id_ (mg g^−1^ min^−1/2^)	0.165	0.376	0.424	0.275	0.602	1.224
*C*_i_ (mg g^−1^)	6.732	3.603	14.91	17.96	9.781	11.37
*R* ^2^	0.5473	0.7487	0.8901	0.9914	0.8620	0.7006

**Boyd model**						
*R* ^2^	0.7871	0.9909	0.9644	0.9469	0.9702	0.9380

a Experimental conditions: pH = 6, dye concentration = 0.02 mmol L^−1^, temp. = 30 °C, adsorbent dosage = 0.67 g L^−1^

#### Pseudo-first order kinetics

The pseudo-first order kinetics model, also called Lagergren's model, can be followed if the plot of ln(*q*_e_ − *q*_*t*_) *versus t* yields a linear line bearing a correlation coefficient (*R*^2^) value equal to unity (Table 3). *q*_*t*_ and *q*_e_ (mg g^−1^) represent the adsorbate amounts adhered onto the amine-CoFeNPs at contact time *t* (min) and at equilibrium, respectively. The slope of Lagergren's plot corresponds to pseudo-first order adsorption rate constant *k*_1_ (min^−1^) while the intercept to ln *q*_e_. For a pseudo-first order model to exist, ln *q*_e_ from experimental data must be equivalent to the intercept of Lagergren's plot.^68^ The Lagergren's equation of the pseudo-first order model is normally valid over the first 20–30 min of the adsorption action, and not for the complete set of contact time range. The results of pseudo-first order kinetics analysis for adsorption of azo dyes onto CoFeNPs1 (Table 4 and Fig. S7a†) and CoFeNPs2 (Table 5 and Fig. S7b†) indicate good linearity but poorer fit of the experimental data to this model compared to the pseudo-second order model.

#### Pseudo-second order kinetics

The kinetics model of pseudo-second order adsorption envisages an adsorption trend over the entire study range and agrees with chemisorption being the rate-limiting stage. A linear pseudo-second order graph between *t*/*q*_*t*_ and *t* provides 1/(*k*_2_*q*_e_^2^) as the intercept and 1/*q*_e_ as the slope (Table 3). *k*_2_ (g mg^−1^ min^−1^) gives the value of pseudo-second order rate constant.^69^ The correlation coefficients for the pseudo-second order kinetics model for each of CoFeNPs1 (Table 4 and Fig. 13a) and CoFeNPs2 (Table 5 and Fig. 13b) were the highest and almost equal to unity. The *q*_e_ (equilibrium adsorption capacity) values computed by pseudo-second order kinetics plots of CoFeNPs1 and CoFeNPs2 were also in very close agreement with the empirical *q*_e_ values contrary to the *q*_e_ values from pseudo-first order plots (Tables 4 and 5), showing a best compliance of the adsorption of anionic azo dyes by CoFeNPs1 and CoFeNPs2 with pseudo-second order kinetics.

#### Elovich kinetics model

The mechanism of activated chemisorption on highly heterogeneous adsorbents is best described by the Elovich equation shown in Table 3. The parameters *a* and *b* in the Elovich equation are computed through the intercept and slope of the straight-line plot between *q*_*t*_ and ln *t*, and they describe the initial adsorption rate (mg g^−1^ min^−1^) and desorption constant (g mg^−1^) associated to the surface coverage extent, respectively.^70^ The *R*^2^ values obtained from the Elovich kinetics plots for CoFeNPs1 (Fig. S8a†) and CoFeNPs2 (Fig. S8b†) were in the range of 0.780–0.966 for CoFeNPs1 (Table 4) and 0.730–0.975 for CoFeNPs2 (Table 5), showing good linearity but poorer fit of the dye adsorption to the Elovich model than the pseudo-second order model.

#### Diffusion kinetics and mechanism

The adsorbate diffusion during the adsorption process can involve four main types of independent mechanisms which may occur simultaneously or sequentially.^71^ These include: diffusion in bulk, film diffusion, intra-particle diffusion, and adsorbate adsorption by active sites on the interior face of the adsorbent. The intra-particle diffusion and Boyd models can be applied to predict the actual diffusion mechanism for the course of adsorption.

Control of the adsorption rate by the intra-particle diffusion phenomenon as the solitary rate-governing step can be confirmed by obtaining a straight-line plot of *q*_*t*_*versus t*^1/2^ passing through the origin (Table 3).^29^ The slope gives the rate constant of intra-particle diffusion (*k*_id_, mg g^−1^ min^−1/2^), whereas the value of the intercept for stage i confers detail of the boundary layer thickness (*C*_i_, mg g^−1^). Fig. 14 provides intra-particle diffusion graphs for the adsorption of anionic dyes by CoFeNPs1 and CoFeNPs2. All plots show two linear regions with different slopes, but no line passes through the origin as *C*_i_ ≥ 2.204 mg g^−1^ (Tables 4 and 5). This indicates at least two major steps involved in the adsorption: the first linear portion is ascribed to dye diffusion from the solution to easily accessible binding sites on the external surface of the amine-CoFeNPs (film or boundary layer diffusion, mass transfer effect), while the second linear region is ascribed to dye diffusion into less accessible internal pores (intra-particle diffusion) until attaining equilibrium.^69^ In general, AO52 exhibits the narrowest boundary layer (lowest *C*_i_) for both amine-CoFeNPs, most probably because of its smallest size and symmetrical (*para*-substituted) structure that can easily penetrate/diffuse into the interior face of NPs following film diffusion, while the intercept (*C*_i_) is greater for other dyes larger in size for which the adsorption is more boundary layer-controlled.^72^

The Boyd model provides information about the slowest step in the course of adsorption. The Boyd plot is a plot between *B*_*t*_ and time *t* (min) (Table 3). The fraction of dye adsorbed onto CoFeNPs at time *t* (*i.e.*, *F* in the expression of *B*_*t*_) can be calculated by the ratio *q*_*t*_/*q*_e_.^15^ The Boyd plots for adsorption of six different anionic azoic dyes onto CoFeNPs1 and CoFeNPs2 are illustrated in Fig. 15, and the related *R*^2^ values (correlation coefficients) are reported in Tables 4 and 5. The Boyd plots are linear with *R*^2^ values of 0.877–0.989 for CoFeNPs1 and 0.787–0.991 for CoFeNPs2; however, they do not cross the origin. Therefore, it is suggested that the external mass transfer, mainly governed by film diffusion, is the rate-controlling mechanism for azo dye removal by CoFeNPs.^73^

### Adsorption isotherms

The most appropriately fitted isotherm model obtained from equilibrium adsorption studies is fundamentally important in designing an optimized adsorption system for dye removal and determining the nature of dye layer coverage on the adsorbent surface. In this regard, the experimental equilibrium data of RO16 adsorption by CoFeNPs1 and CoFeNPs2 at optimized conditions (pH 4, temperature 30 °C and adsorbent dosage 0.67 g L^−1^) for seven different initial dye concentrations (12.4, 18.5, 24.7, 30.9, 37.1, 43.2 and 49.4 mg L^−1^) were applied to fit the Langmuir and Freundlich isotherm models. The Langmuir isotherm model applies when saturated monolayer coverage of adsorbate molecules occurs (without lateral interaction between adsorbed molecules) on a homogeneous adsorbent surface of invariable energy, whereas the Freundlich isotherm model assumes multilayer adsorption of adsorbate on a heterogeneous adsorbent surface with different energy sites involving mutual interactions between adsorbed species.^15^ The expressions of the linear form of the Langmuir and Freundlich adsorption isotherms are given below as eqn (1) and (2), respectively.

1 *C*_e_/*q*_e_ = 1/(*q*_max_*K*_L_) + (1/*q*_max_)*C*_e_

2 log *q*_e_ = log *K*_F_ + (1/*n*)log *C*_e_

**Fig. 16:**
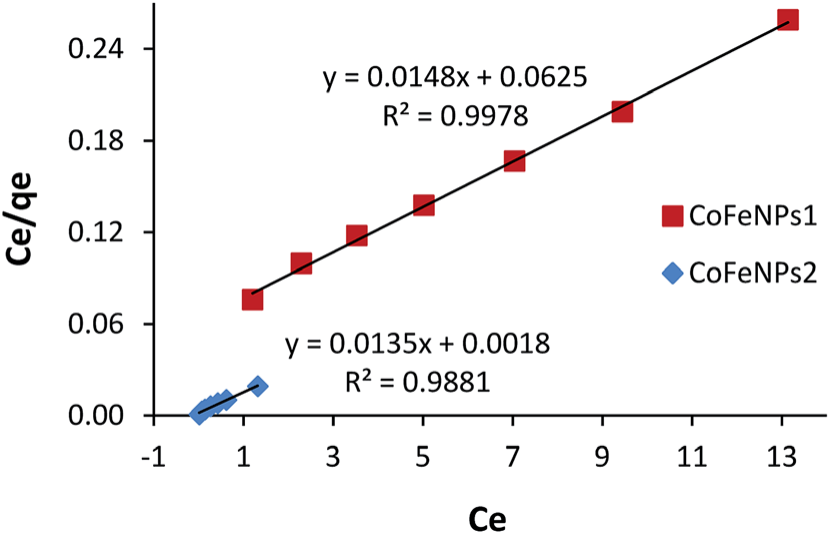
Langmuir adsorption isotherm for the removal of RO16 by CoFeNPs1 and CoFeNPs2.

*C*_e_ is the equilibrium concentration of adsorbate in the bulk liquid phase (mg L^−1^) and *q*_e_ is the dye uptake per unit mass of adsorbent (mg g^−1^) at a constant temperature. The constants *q*_max_ and *K*_L_ of the Langmuir isotherm describe the maximum adsorption capacity (mg g^−1^) of the adsorbent for a monolayer and heat of adsorption (L mg^−1^), respectively, whereas the Freundlich constants *K*_F_ (mg^1−1/*n*^ L^1/*n*^ g^−1^) and *n* (heterogeneity factor, unitless) are a measure of adsorption capacity and strength of adsorption, respectively.^29^ The adsorption parameters and correlation coefficients (*R*^2^) computed from the Langmuir and Freundlich isotherm plots (Fig. 16 and S9†) for RO16 adsorption onto CoFeNPs1 and CoFeNPs2 are provided in Table 6.

**Table 6:** Adsorption isotherm parameters for RO16 adsorption onto CoFeNPs1 and CoFeNPs2^a^

	Langmuir isotherm				Freundlich isotherm		
	*q*_max_ (mg g^−1^)	*K*_L_ (L mg^−1^)	*R*_L_	*R* ^2^	*K*_F_ (mg^1−1/*n*^ L^1/*n*^ g^−1^)	*n*	*R* ^2^
CoFeNPs1	67.57	0.237	0.255–0.079	0.998	15.26	1.993	0.982
CoFeNPs2	74.07	7.500	0.011–0.003	0.988	65.54	3.254	0.9852

a Experimental conditions: pH = 4, temp. = 30 °C, adsorbent dosage = 0.67 g L^−1^.

### Thermodynamics of the adsorption of amine-CoFeNPs

**Fig. 17:**
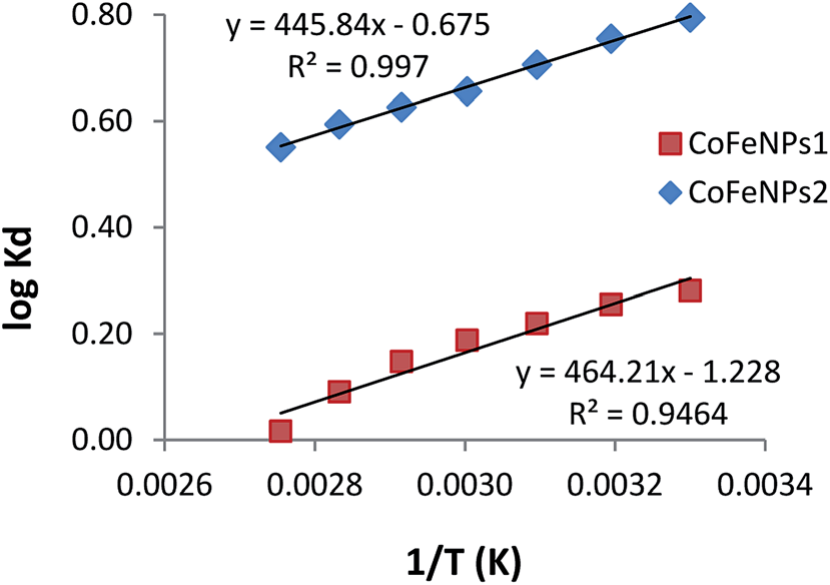
van't Hoff plots of adsorption of RO16 onto CoFeNPs1 and CoFeNPs2.

Thermodynamics studies were conducted to determine the influence of temperature and calculate the thermodynamic parameters (Δ*H*°, Δ*S*° and Δ*G*°) for dye removal by CoFeNPs1 and CoFeNPs2. Selecting RO16 dye for this purpose, adsorption experiments were carried out at seven different temperatures (303, 313, 323, 333, 343, 353 and 363 K) keeping other variables constant (pH 6, RO16 concentration 0.02 mmol L^−1^ and adsorbent dosage 0.67 g L^−1^). The changes in entropy (Δ*S*°) and enthalpy (Δ*H*°), given in Table 7, were computed using the van't Hoff regression plot of log *K*_d_*versus* 1/*T* (Fig. 17) based on van't Hoff eqn (3).^74^ The standard Gibbs free energy change (Δ*G*°) during adsorption at various temperatures was calculated from eqn (5).

3 log *K*_d_ = (Δ*S*°/2.303*R*) – (Δ*H*°/2.303*RT*)

4 *K*_d_ = *q*_e_/*C*_e_

5 Δ*G*° = Δ*H*° − *T*Δ*S*°

where *K*_d_ is the distribution coefficient for adsorption (L g^−1^), *q*_e_ is the RO16 amount adsorbed onto amine-CoFeNPs at equilibrium (mg g^−1^), *C*_e_ is the RO16 concentration at equilibrium in the liquid phase (mg L^−1^), *R* is the universal gas constant (8.314 J mol^−1^ K^−1^), and *T* is the absolute temperature (K).

**Table 7:** Parameters of thermodynamics of RO16 adsorption onto CoFeNPs1 and CoFeNPs2

	*T* (K)	*K*_d_ (L g^−1^)	Δ*G*° (kJ mol^−1^)	Δ*H*° (kJ mol^−1^)	Δ*S*° (J mol^−1^ K^−1^)	*R* ^2^
CoFeNPs1	303	1.910	−1.765	−8.888	−23.51	0.946
	313	1.797	−1.530			
	323	1.657	−1.295			
	333	1.541	−1.059			
	343	1.405	−0.824			
	353	1.232	−0.589			
	363	1.038	−0.354			
CoFeNPs2	303	6.232	−4.622	−8.536	−12.92	0.997
	313	5.684	−4.493			
	323	5.079	−4.364			
	333	4.524	−4.179			
	343	4.225	−4.109			
	353	3.925	−4.013			
	363	3.556	−3.828			

The adsorption of RO16 onto amine-CoFeNPs is exothermic as the Δ*H*° values are negative, also confirmed by the decreased RO16 removal efficiency (from 56.0 to 40.9% and 80.6% to 70.3% by CoFeNPs1 and CoFeNPs2, respectively) on increasing the temperature from 303 to 363 K. The negative Δ*S*° values suggest small randomness at the solid–liquid interface during RO16 adsorption onto amine-CoFeNPs, ascribed to trivial structural changes in the adsorbate and adsorbent. The negative Δ*G*° values at various studied temperatures suggest thermodynamically favorable and spontaneous adsorption of RO16 onto amine-CoFeNPs.^8^ However, an increase in temperature decreases the degree of spontaneity and thermodynamic feasibility, as shown by higher Δ*G*° values at higher temperatures. The small Δ*G*° values between 0 and −20 kJ mol^−1^ identify the adsorption process of RO16 by amine-CoFeNPs predominantly as physisorption.^75^

### Reusability of amine-CoFeNPs

The regeneration and appropriate reusability of an adsorbent increases its industrial significance and economic value. Therefore, the recovery/desorption of dye (RO16) from spent CoFeNPs1 and CoFeNPs2 was studied in detail using four different effluents (1 M HCl, 2 M NaOH, methanol, and 9 : 1 (v/v) methanol : acetic acid mixture) at 30 °C and adsorbent dosage 0.67 g L^−1^.

**Fig. 18:**
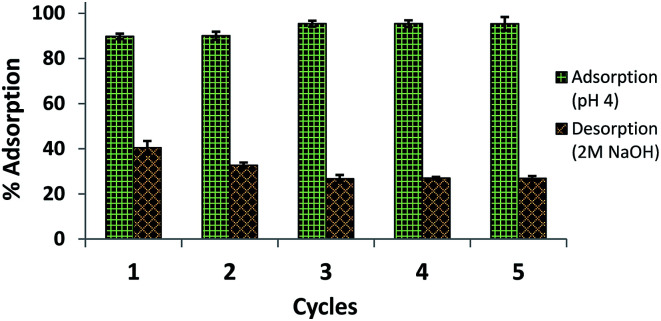
Reusability of CoFeNPs1 for 5 cycles using 2 M NaOH [conditions: initial RO16 concentration 0.02 mmol L^−1^, adsorbent dosage 0.67 g L^−1^, temp. 30 °C, contact time 2 h].

**Fig. 19:**
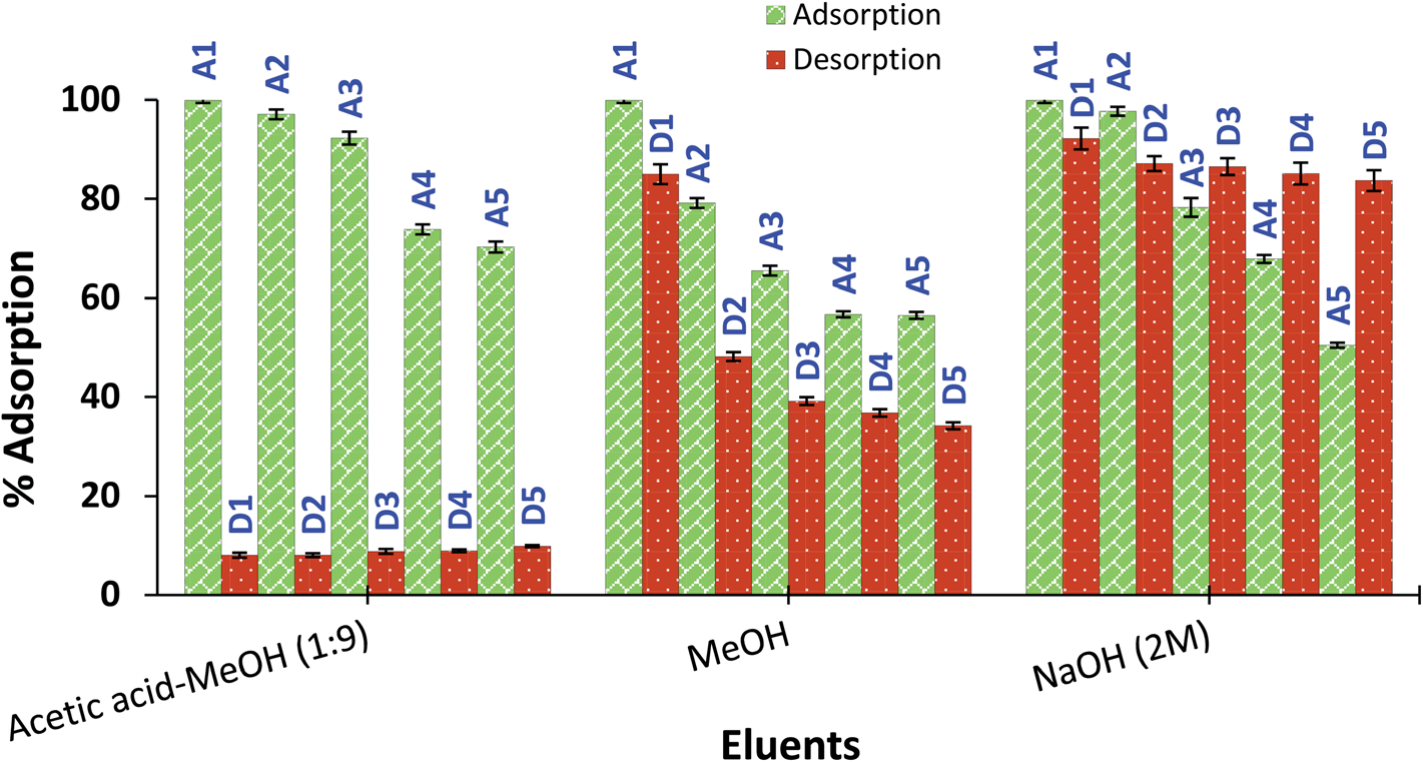
Reusability of CoFeNPs2 for 5 cycles using three different eluents. A represents RO16 adsorption (at pH 4), and D represents desorption in the eluent [conditions: initial RO16 concentration 0.02 mmol L^−1^, adsorbent dosage 0.67 g L^−1^, temp. 30 °C, contact time 2 h].

CoFeNPs1 showed significant desorption of RO16 in NaOH within 1.5 h and can be conveniently reused for at least five successive adsorption–desorption cycles with enhanced percent adsorption and desorption in successive runs (Fig. 18). Such enhanced adsorption in successive runs was also observed in previous reusability studies of a histidine–magnetite NPs adsorbent with Acid Black 1 dye.^15^ The NaOH concentration (1 M or 2 M) did not significantly affect the percent desorption from CoFeNPs1. MeOH, HCl (1 M) and MeOH/CH_3_COOH mixture (9 : 1) gave no desorption from CoFeNPs1; instead, HCl and the MeOH/CH_3_COOH mixture caused slow dissolution of CoFeNPs1 after a day. In contrast, spent CoFeNPs2 showed the highest desorption (91.96%) and excellent reusability with the MeOH/CH_3_COOH mixture within 2 h (Fig. 19). CoFeNPs2 also showed slight desorption with MeOH (15%) and 2 M NaOH (8%) in the first desorption run, which interestingly increased up to 66% and 16%, respectively, until the 5th desorption run. Even yielding relatively poor desorption by MeOH and 2 M NaOH, CoFeNPs2 showed >50% adsorption (at pH 4) until the 5th adsorption run. Desorption of RO16 in basic medium from both CoFeNPs1 and CoFeNPs2, compared to no desorption in acidic medium, is probably due to the CoFeNPs gaining negative surface charge, hence causing electrostatic repulsion between the anionic dyes and adsorbent in an alkaline environment, favoring desorption in a basic medium. This also confirms the existence of electrostatic attraction between the amine-CoFeNPs and anionic dyes as suggested in the dye removal mechanism. However, the differences in the relative percent desorption of CoFeNPs1 and CoFeNPs2 in a base is probably because of the extent of other interactions involved in adsorption. The highest desorption of RO16 in MeOH/CH_3_COOH (9 : 1) from used CoFeNPs2 is expected mainly due to π–π interaction of CH_3_COOH with the dye, breaking the strong hydrophobic interactions between CoFeNPs2 and the dye, and hence favoring desorption. Slight desorption from used CoFeNPs2 in MeOH alone is probably because of some dissolution of RO16 in the polar organic solvent. Such dye dissolution in MeOH is not obvious for CoFeNPs1, probably due to stronger hydrophilic interactions among CoFeNPs1 and RO16. Hence, all the results of the desorption studies strongly corroborate the suggested mechanism of interaction of the amine-CoFeNPs with anionic dyes. Furthermore, amine-CoFeNPs as adsorbents exhibit excellent reusability.

## Conclusions

This study describes the successful development and characterization of two types of amine-functionalized magnetic CoFe_2_O_4_ nanoparticles (*i.e.*, CoFeNPs1 and CoFeNPs2, functionalized with hydrazine and dodecylamine, respectively) to capitulate the removal of six structurally different anionic azo dyes from their aqueous solutions and explore their adsorptive application. This study indicates that structural differences in the functionalized amine affect various physical and chemical features of the CoFeNPs (such as their particle size, thermal stability/degradation behavior, surface charge, and dye adsorption efficiency/removal mechanism) to a certain extent. As a result, CoFeNPs2 revealed relatively larger size, more positive surface charge, lower thermal stability with pronounced textural changes during thermal degradation, and better adsorption efficiency for all six dyes, compared to CoFeNPs1. The degree of dye adsorption of the amine-CoFeNPs shows a strong relationship (positive for CoFeNPs2 and negative for CoFeNPs1) with various structural parameters of the dyes, such as their size, charge, complexity and hydrophobicity. The promising dye removal ability of amine-CoFeNPs within a short time compared to other adsorbents, even at unoptimized conditions, warrants further research on CoFeNPs as adsorbents to treat dye-contaminated solutions or wastes. A study of simultaneous dye removal from a mixture of all dyes is recommended to investigate the effect of interference of a dye in the removal of other dyes. The electronic and IR spectra revealed aggregation of some dyes in addition to adsorption on CoFeNPs2, owing to strong hydrophobic linkages between the coated surfactant and some dyes. Such aggregations, causing spectral shifts in the dye absorption bands, should be studied in detail to find additional roles of CoFeNPs2 in optical applications. Different amine-CoFeNPs could be selectively applied for efficient, economic and rapid treatment of industrial waste containing certain azo dyes, considering their structural features.

## Conflicts of interest

There are no conflicts to declare.

## Supplementary Material

RA-010-C9RA07686G-s001
