# Supplementary material for: Anionic azo dyes removal from water using amine-functionalized cobalt–iron oxide nanoparticles: a comparative time-dependent study and structural optimization towards the removal mechanism
Source: RSC Adv. 2020 Jan 3;10(2):1021–41. doi: 10.1039/c9ra07686g (PMC9048384; doi:10.1039/c9ra07686g)
Supplement: RA-010-C9RA07686G-s001 [file RA-010-C9RA07686G-s001.pdf]

Electronic Supplementary Material (ESI) for RSC Advances.
This journal is © The Royal Society of Chemistry 2019

RSC Advances

ELECTRONIC SUPPLEMENTARY INFORMATION

Anionic Azo Dyes Removal From Water using Amine-Functionalized Cobalt-Iron Oxide Nanoparticles: A Comparative Time-Dependant Study and Structural Optimization towards Removal Mechanism

Qurrat-ul-Ain,[a]* Sumaira Khurshid,[b] Zarnab Gul,[a] Jaweria Khatoon,[b] Muhammad Raza Shah,[c] Irum Hamid,[a] Iffat Abdul Tawab Khan[b] and Fariha Aslam[c]

[a] *Department of Chemistry, Faculty of Science, University of Karachi, Karachi-75270, Pakistan*
Email: qurrat_chem@uok.edu.pk; Fax: +92.21.99261330; Tel: +92.21.99261300
[b] *Department of Chemistry, Federal Urdu University of Arts, Science and Technology, Gulshan-e-Iqbal Campus, Karachi-75300, Pakistan*
[c] *H. E. J. Research Institute of Chemistry, ICCBS, University of Karachi, Karachi 75270, Pakistan*
Email: raza.shah@iccs.edu

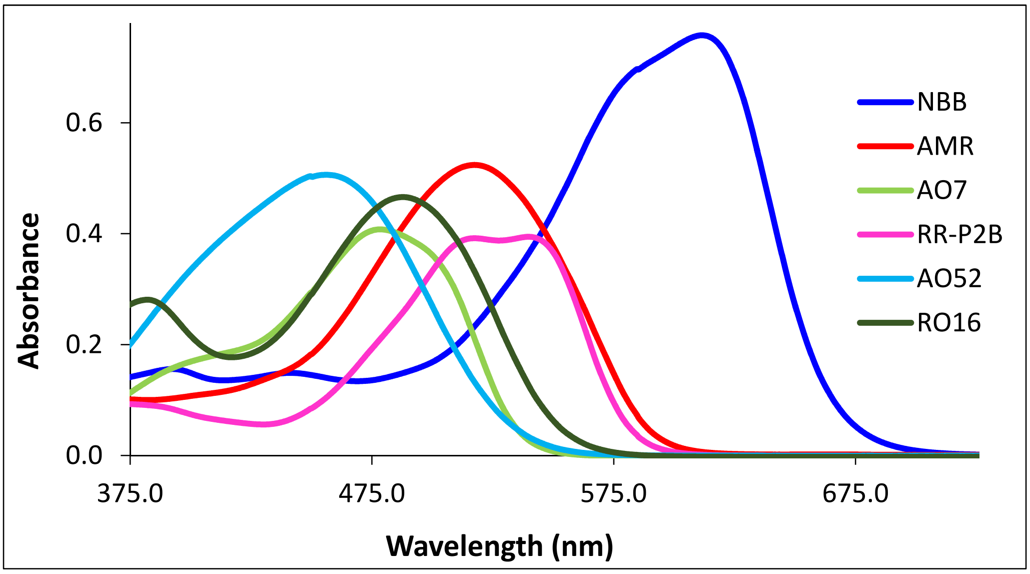

Fig. S1 Overlayed electronic spectra of aqueous NBB, AMR, AO7, RR-P2B, AO52 and RO16 (initial dye concentration = 0.02 mmol L^{-1}, temp. = 30°C, pH = 6).

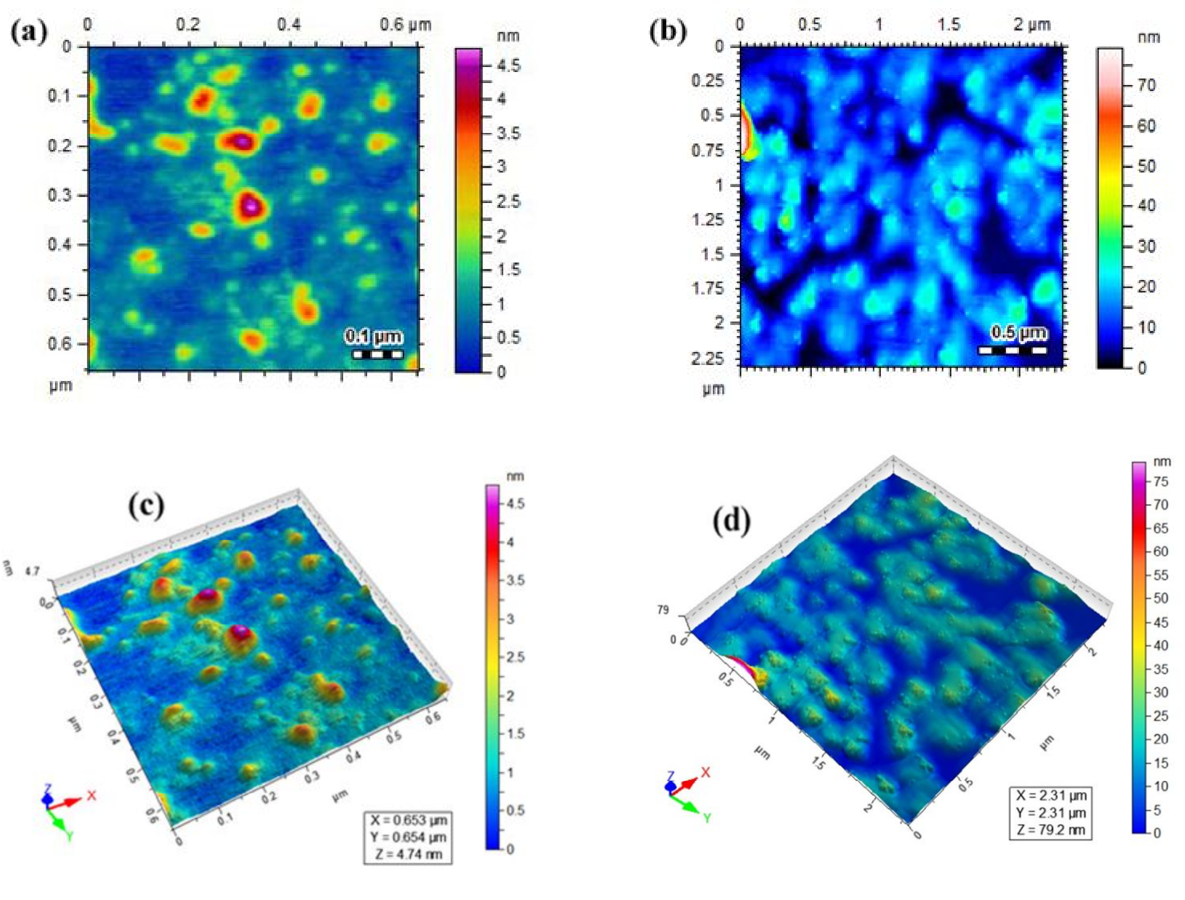

CoFeNPs1 **CoFeNPs2**

Fig. S2 AFM analysis of amine-functionalized CoFeNPs1 (left) and CoFeNPs2 (right). (a, b) two-dimensional surface images, and (c, d) three-dimensional surface images.

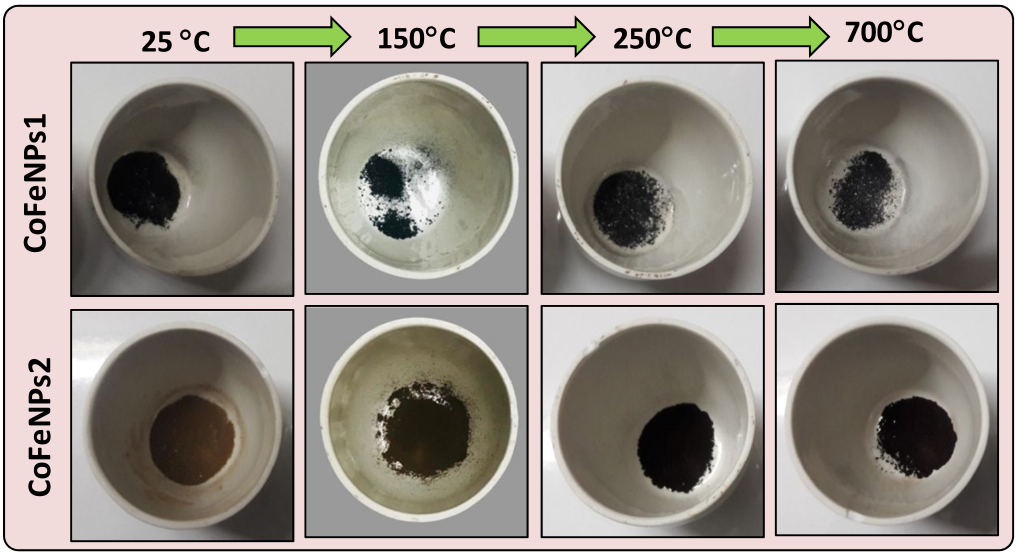

Fig. S3 Color and textural changes observed during aerobic heating of CoFeNPs1 and CoFeNPs2.

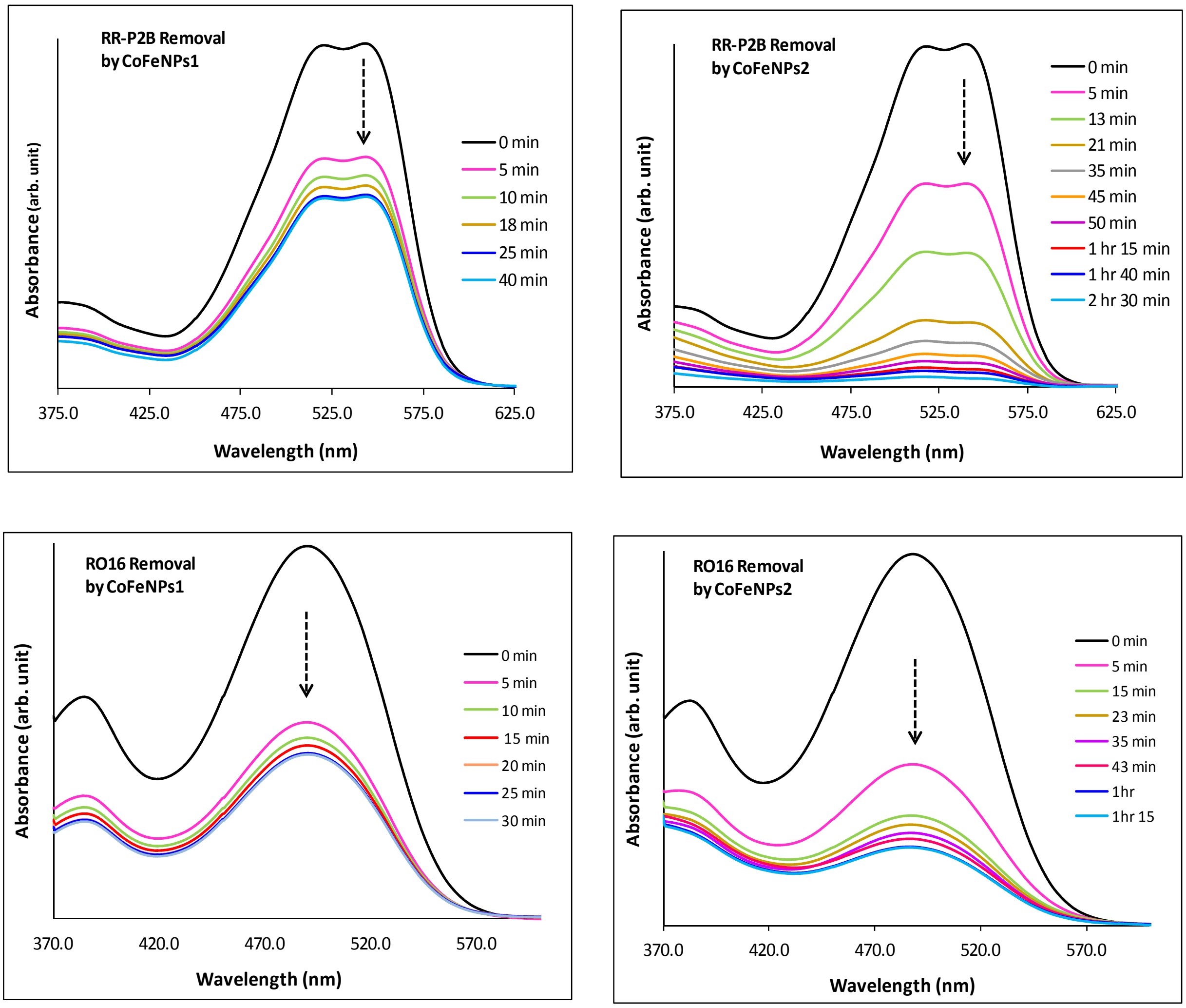

Fig. S4 Comparative electronic spectra of removal of RR-P2B and RO16 at various time intervals by CoFeNPs1 (left) and CoFeNPs2 (right).

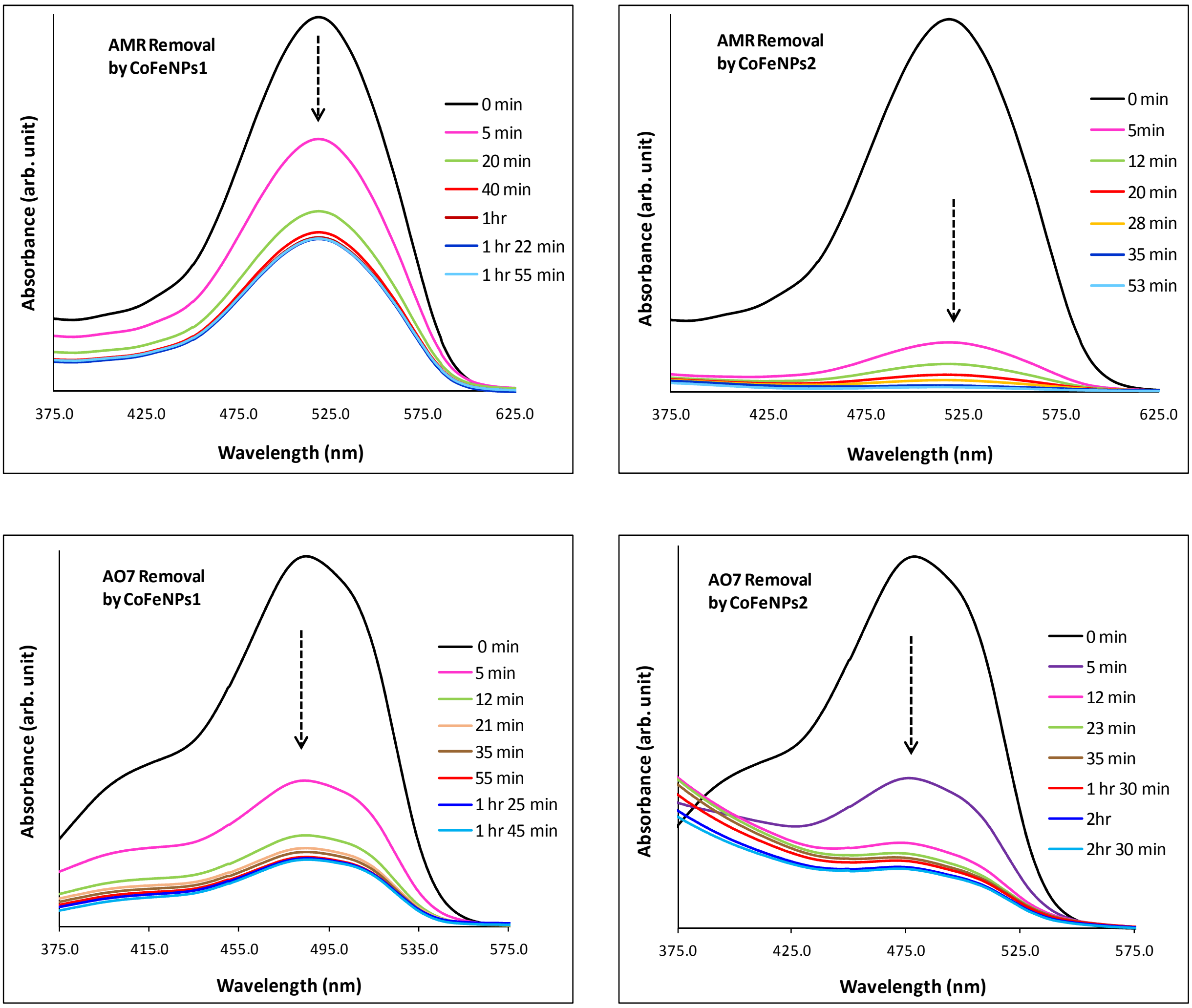

Fig. S5 Comparative electronic spectra of removal of AMR and AO7 at various time intervals by CoFeNPs1 (left) and CoFeNPs2 (right).

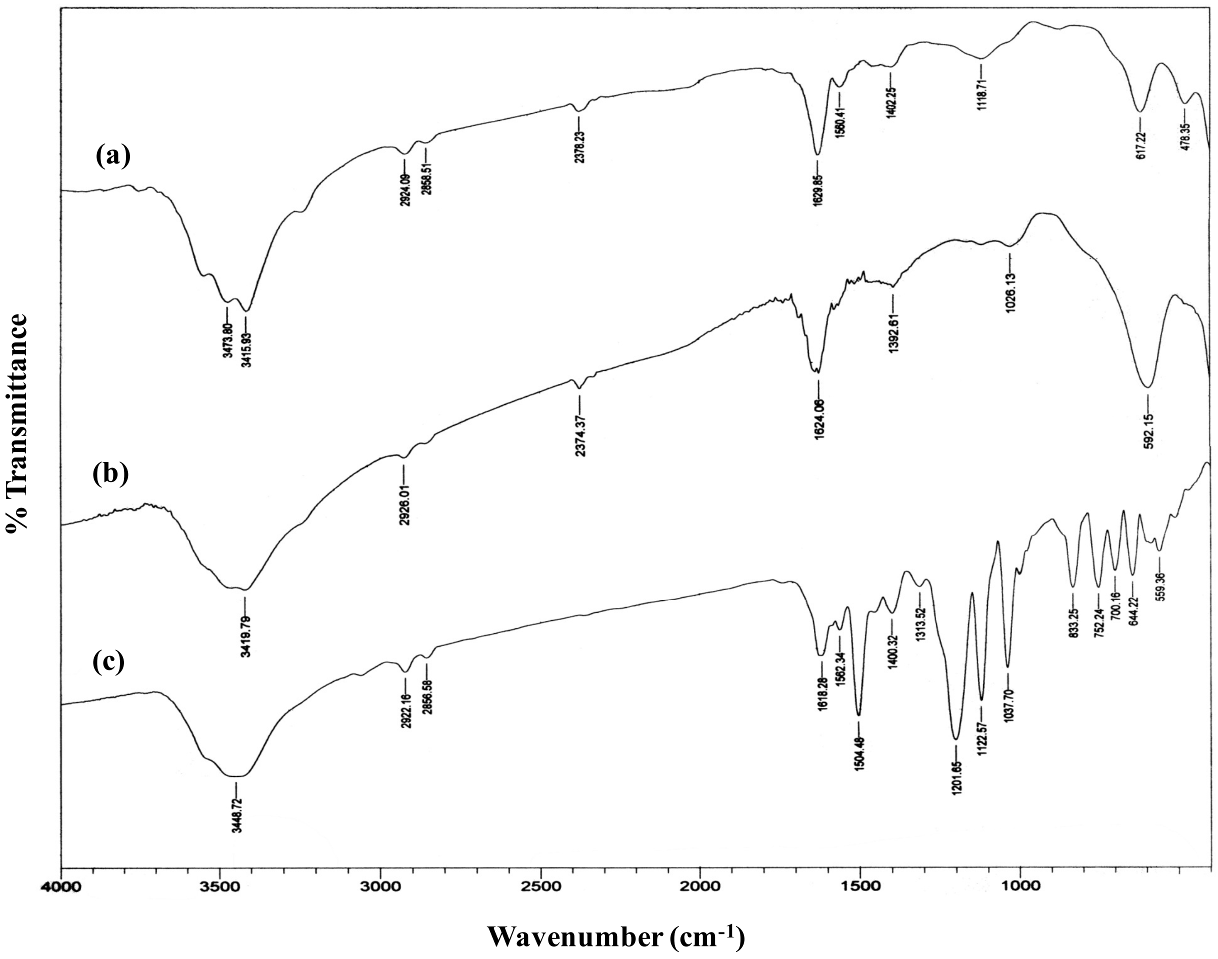

Fig. S6 FT-IR spectrum of (a) AO7-treated CoFeNPs1, (b) AO7-treated CoFeNPs2 and (c) control AO7 dye.

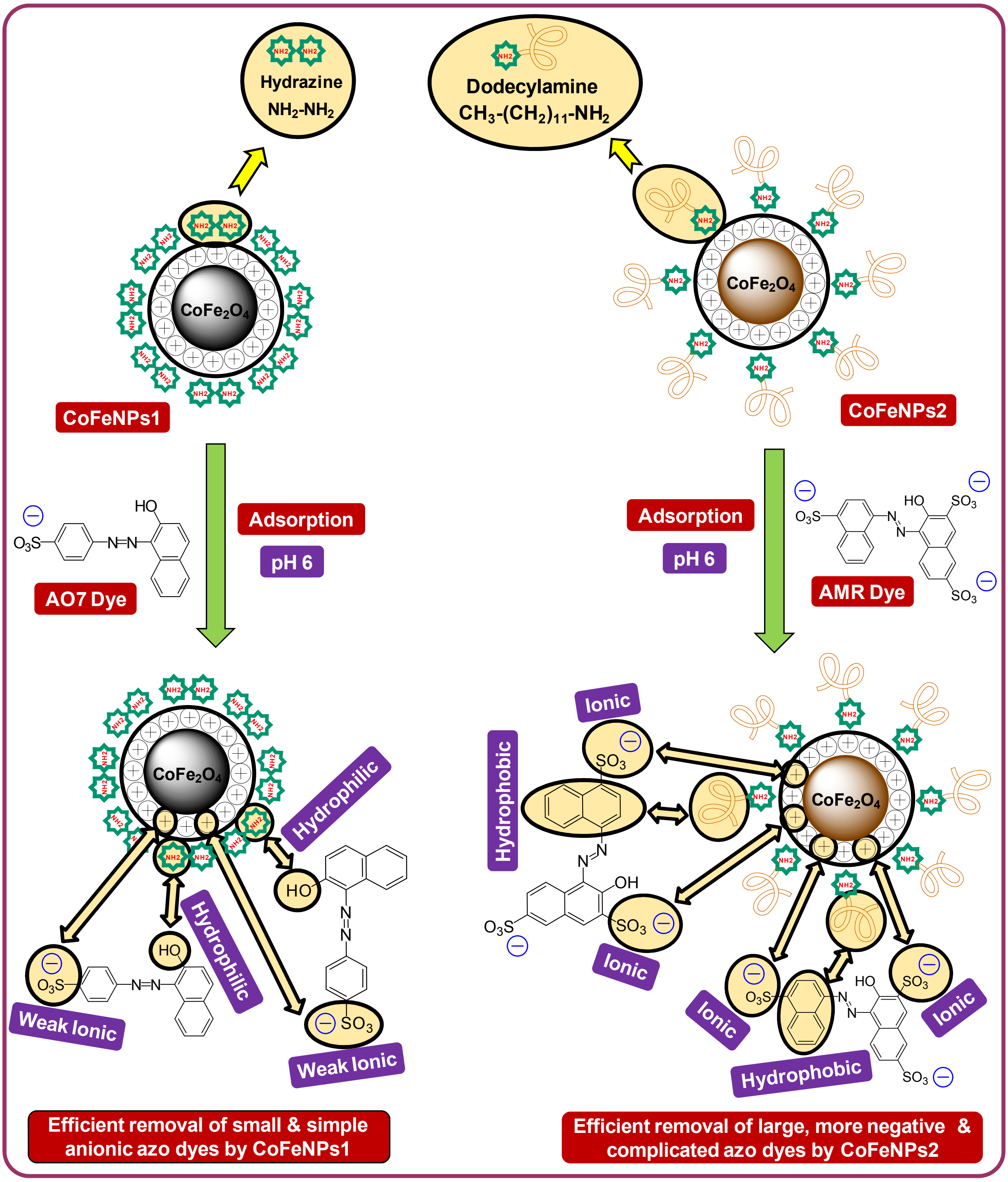

Scheme S1 Probable mechanism of removal of anionic azo dyes by amine-functionalized CoFeNPs (AO7 selected for CoFeNPs1 and AMR selected for CoFeNPs2) showing structural effects and all plausible interactions between NPs and dyes.

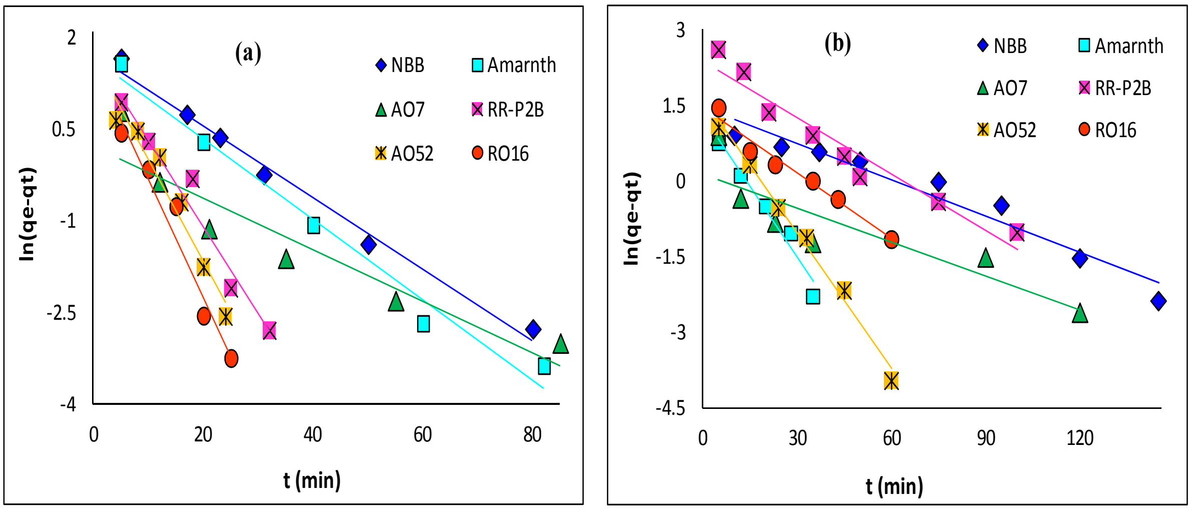

Fig. S7 Pseudo-first order plots of kinetics for the adsorption of anionic azo dyes onto (a) CoFeNPs1 and (b) CoFeNPs2.

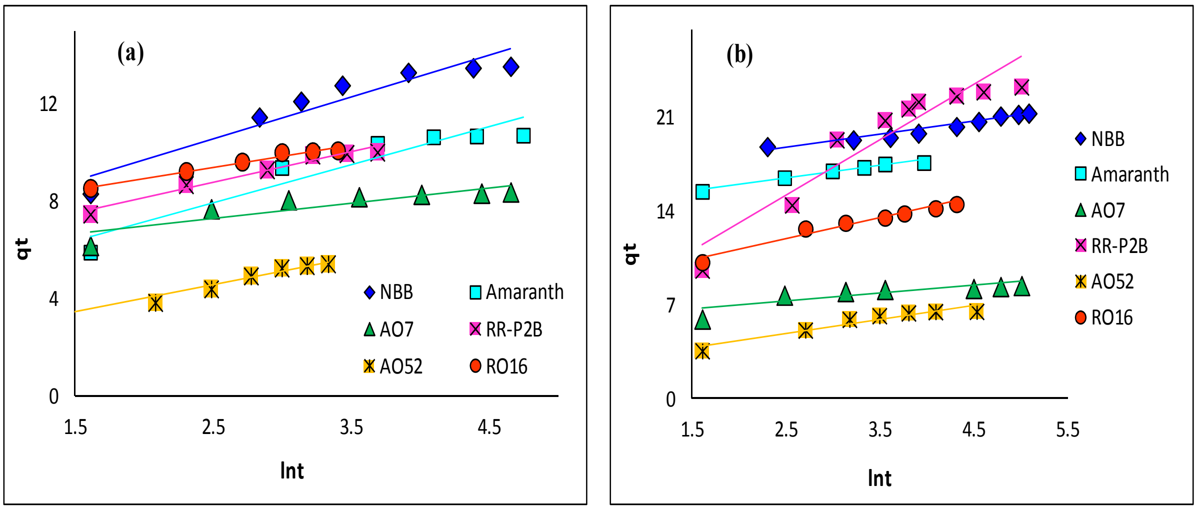

Fig. S8 Elovich plots of kinetics for the adsorption of anionic azo dyes onto (a) CoFeNPs1 and (b) CoFeNPs2.

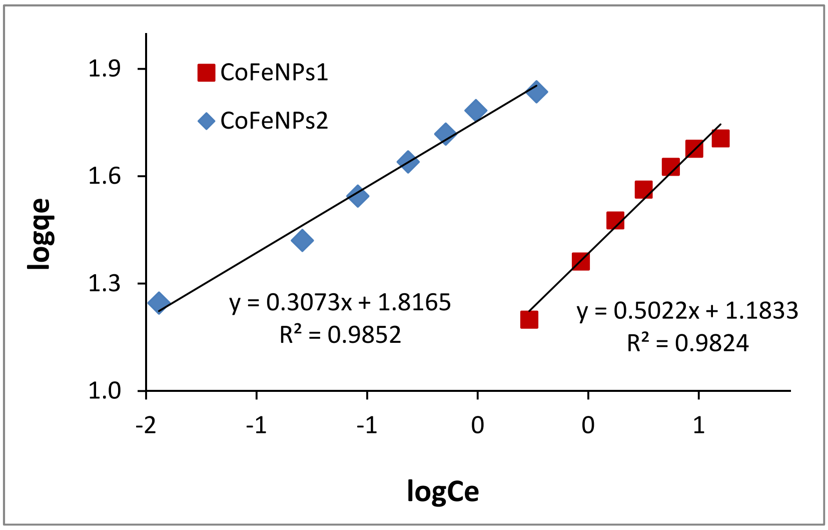

Fig. S9 Freundlich adsorption isotherm for the removal of RO16 by CoFeNPs1 and CoFeNPs2.
